# Co-occurrence Analysis of Microbial Taxa in the Atlantic Ocean Reveals High Connectivity in the Free-Living Bacterioplankton

**DOI:** 10.3389/fmicb.2016.00649

**Published:** 2016-05-06

**Authors:** Mathias Milici, Zhi-Luo Deng, Jürgen Tomasch, Johan Decelle, Melissa L. Wos-Oxley, Hui Wang, Ruy Jáuregui, Iris Plumeier, Helge-Ansgar Giebel, Thomas H. Badewien, Mascha Wurst, Dietmar H. Pieper, Meinhard Simon, Irene Wagner-Döbler

**Affiliations:** ^1^Group Microbial Communication, Helmholtz-Center for Infection ResearchBraunschweig, Germany; ^2^UMR 7144 - Sorbonne Universités, UPMC Univ Paris 06Roscoff, France; ^3^Centre National de la Recherche Scientifique, UMR 7144Roscoff, France; ^4^Group Microbial Interactions and Processes, Helmholtz-Center for Infection ResearchBraunschweig, Germany; ^5^Biology of Geological Processes, Institute for Chemistry and Biology of the Marine Environment, University of OldenburgOldenburg, Germany

**Keywords:** co-occurrence, bacteria-bacteria interactions, phytoplankton-bacteria interactions, next generation sequencing, Atlantic Ocean, bacterioplankton, 16S rRNA analysis, network analysis

## Abstract

We determined the taxonomic composition of the bacterioplankton of the epipelagic zone of the Atlantic Ocean along a latitudinal transect (51°S–47°N) using Illumina sequencing of the V5-V6 region of the 16S rRNA gene and inferred co-occurrence networks. Bacterioplankon community composition was distinct for Longhurstian provinces and water depth. Free-living microbial communities (between 0.22 and 3 μm) were dominated by highly abundant and ubiquitous taxa with streamlined genomes (e.g., SAR11, SAR86, OM1, *Prochlorococcus*) and could clearly be separated from particle-associated communities which were dominated by Bacteroidetes, Planktomycetes, Verrucomicrobia, and Roseobacters. From a total of 369 different communities we then inferred co-occurrence networks for each size fraction and depth layer of the plankton between bacteria and between bacteria and phototrophic micro-eukaryotes. The inferred networks showed a reduction of edges in the deepest layer of the photic zone. Networks comprised of free-living bacteria had a larger amount of connections per OTU when compared to the particle associated communities throughout the water column. Negative correlations accounted for roughly one third of the total edges in the free-living communities at all depths, while they decreased with depth in the particle associated communities where they amounted for roughly 10% of the total in the last part of the epipelagic zone. Co-occurrence networks of bacteria with phototrophic micro-eukaryotes were not taxon-specific, and dominated by mutual exclusion (~60%). The data show a high degree of specialization to micro-environments in the water column and highlight the importance of interdependencies particularly between free-living bacteria in the upper layers of the epipelagic zone.

## Introduction

Microorganisms play an essential role in all terrestrial and aquatic ecosystems. Their activity directly influences biogeochemical cycles of essential elements like carbon, nitrogen, and sulfur (Azam and Malfatti, 2007; Falkowski et al., 2008; Zehr and Kudela, 2011; Moran et al., 2012), and thus has a tremendous effect on the whole planet. In the ocean, prokaryotes (bacteria and archaea) are the most abundant living organisms, with an average of 5 × 10^5^ cells per milliliter of sea water on the continental shelf but also in the upper 200 m of the oceanic water column (Whitman et al., 1998). However, this number refers only to the domains of bacteria and archaea without taking into account eukaryotic microorganisms (Caron et al., 2012; de Vargas et al., 2015), viruses (Suttle, 2005; Brum et al., 2015) or the ultra-small bacteria discovered recently (Brown et al., 2015). It is therefore easy to picture how crowded a milliliter of seawater is. The high number of microorganisms (archaea, bacteria, eukaryotes) and viruses per milliliter of seawater and their long evolutionary history (Cavalier-Smith, 2006) naturally forces the coexistence, interaction, and possibly co-evolution of microorganisms in the same spatial niche.

Within a milliliter of seawater, spatial niches are generally defined according to the pore size of the filter that is used to collect microorganisms. Filters with larger pore-size (generally larger than 3 μm) collect particles, micro-eukaryotes, and attached or ingested bacteria, while the truly planktonic or free-living bacteria are typically captured on 0.1–0.22 μm filters. However, the separation of the bacterioplankton according to the filter size does not strictly reflect bacterial lifestyle (Hartmann et al., 2013; Padilla et al., 2015), nevertheless, serial filtration represents a valid method that permits to investigate bacterioplankton communities from different size fractions of the plankton (Rusch et al., 2007; Sunagawa et al., 2015; Salazar et al., 2016). Bacteria collected on bigger size fractions (>3 μm) are presumably physically attached to particles and are often chemotactic (Stocker and Seymour, 2012). Free-living bacterial communities are dominated by cosmopolitan oligotrophic taxa with streamlined genomes such as the ubiquitous clade SAR11 (Morris et al., 2002; Grote et al., 2012; Morris R. M. et al., 2012; Giovannoni et al., 2014), while bacteria associated to particles composed of living and decaying phytoplankton and zooplankton as well as fecal pellets and cellular debris (Herndl and Reinthaler, 2013) have been described as copiotrophic. Those tend to have larger genomes with a broad spectrum of metabolic capabilities and are often cultivatable (Allen et al., 2013; Giovannoni et al., 2014). Moreover, many genes in their genomes are devoted to competition (Dang and Lowell, 2016).

Interactions within planktonic communities have been hypothesized to account for an important part of the observed diversity (Fuhrman et al., 2015). Examples for physical mutualistic symbioses in the ocean bacterioplankton have been discovered. For example, the uncultivated diazotroph cyanobacterium UCYN-A lives in an obligate symbiosis with prymnesiophyte (eukaryotic microalga), fixing and translocating nitrogen for the microalgae, which transfers photosynthetically fixed carbon in return (Thompson et al., 2012; Krupke et al., 2015). SAR11, the most abundant marine clade, is critically dependent on reduced sulfur compounds like DMSP (Dimethylsulfoniopropionate) provided by marine algae (Tripp et al., 2008) as well as on vitamins provided by other members of the microbial community (Carini et al., 2014). B vitamins and in particular B12 are essential cofactors of many enzymes, yet <40% of the sequenced genomes reviewed in Sañudo-Wilhelmy et al. (2014) harbored the genetic machinery to fully synthesize cobalamin demonstrating a widespread auxotrophy for it.

It is now possible to resolve the community structure of marine plankton with a resolution high enough to construct co-occurrence networks of OTUs. Such networks can be used to develop hypotheses that can then be verified experimentally, as in the *Tara* Ocean study (Lima-Mendez et al., 2015) and also be used to simulate the effect of species extinction (Peura et al., 2015). Until now, co-occurrence networks have mainly been constructed from time-series data to observe the effect of season, daily variability, and plankton blooms. Time-series data from oceanographic long-term observatories (BATS, SPOT, HOT, and the English Channel) have shown an unexpected resilience of bacterioplankton communities that have largely recurrent composition in the seasonal cycle, while varying considerably on a daily timescale (Fuhrman et al., 2015). Top-down controls on the microbial community through viruses has been investigated (Chow et al., 2014), and from a time-lagged relationship between upper and deeper water layers, it was hypothesized that the downward sinking of particles is one of the determinants of bacterioplankton community structure in deeper water layers (Cram et al., 2015b). Interestingly, environmental parameters, although possibly inducing phytoplankton blooms, were negligible in determining plankton community structure during the weeks that the succession induced by the bloom continued, suggesting that interactions within the community played a much more important role than previously thought (Needham and Fuhrman, 2016). Similarly, the analysis of the *Tara* Oceans dataset demonstrated that environment and geography explained up to 18% of community variation. Using a random forest approach it was shown that 95% of the models based on OTU composition alone were more accurate in explaining variation than models incorporating additionally environmental data (Lima-Mendez et al., 2015).

As far as we know the *Tara* Oceans study is the only one that uses such a holistic network approach on samples covering a geographical range in the ocean, and not a time-series on a fixed location. The network was useful in discovering new and globally important interactions (Lima-Mendez et al., 2015) and was also used to start quantifying and predicting the biological carbon pump (Guidi et al., 2016). Here, we used the network approach to infer co-occurrences of bacteria and photosynthetic micro-eukaryotic in the Atlantic Ocean. The samples were derived from 27 stations across almost the entire Atlantic Ocean (51°S–47°N). For each station, the epipelagic zone (20–200 m) was sampled with an average of five depths per station. Water samples were fractionated into three size classes. Thus, we were able to compare free-living to particle attached communities throughout the transect. Sinking particles export biomass from the euphotic zone to the deep ocean and thus have an important role for the biological carbon pump (Herndl and Reinthaler, 2013). Community composition was investigated by Illumina sequencing of the V5-V6 region of the 16S rRNA gene of bacteria and of algal chloroplasts, thus providing a fairly extensive picture of the co-occurring phototrophic microalgae. We have previously reported a taxonomy independent analysis of the data, based on OTU assignment only. We could show that alpha-diversity does not increase in the tropical regions and has a non-linear relationship with temperature (Milici et al., 2016b). The biogeographical parameters shaping beta-diversity were subsequently analyzed, and we could show that the distance-decay relationship does not hold on a global scale; communities separated by almost 12,000 km across the ocean were most similar to each other; Longhurstian province, depth within the epipelagic zone and the composition of co-occurring micro-eukaryotes accounted for a large fraction of beta-diversity (Milici et al., 2016a). Interestingly, all of those findings applied similarly to bacteria from all three size fractions of the bacterioplankton, independent from the composition of the communities themselves. Here we now describe the taxonomic composition of those samples, showing that Longhurstian oceanographic provinces and depth layers of the epipelagic zone of the Atlantic Ocean enrich for different taxa. Microbial taxa living on particles were also clearly different from those of free-living ones. Certain lineages from almost all phyla detected here were enriched on particles, and certain phyla were exclusively found on them. We then combined the 369 communities in our study according to depth and calculated co-occurrence networks for free-living and particle-associated communities. We found a much higher number of co-occurrences in the free-living communities when compared to the particle associated communities.

## Materials and methods

### Sampling

**Figure 1:**
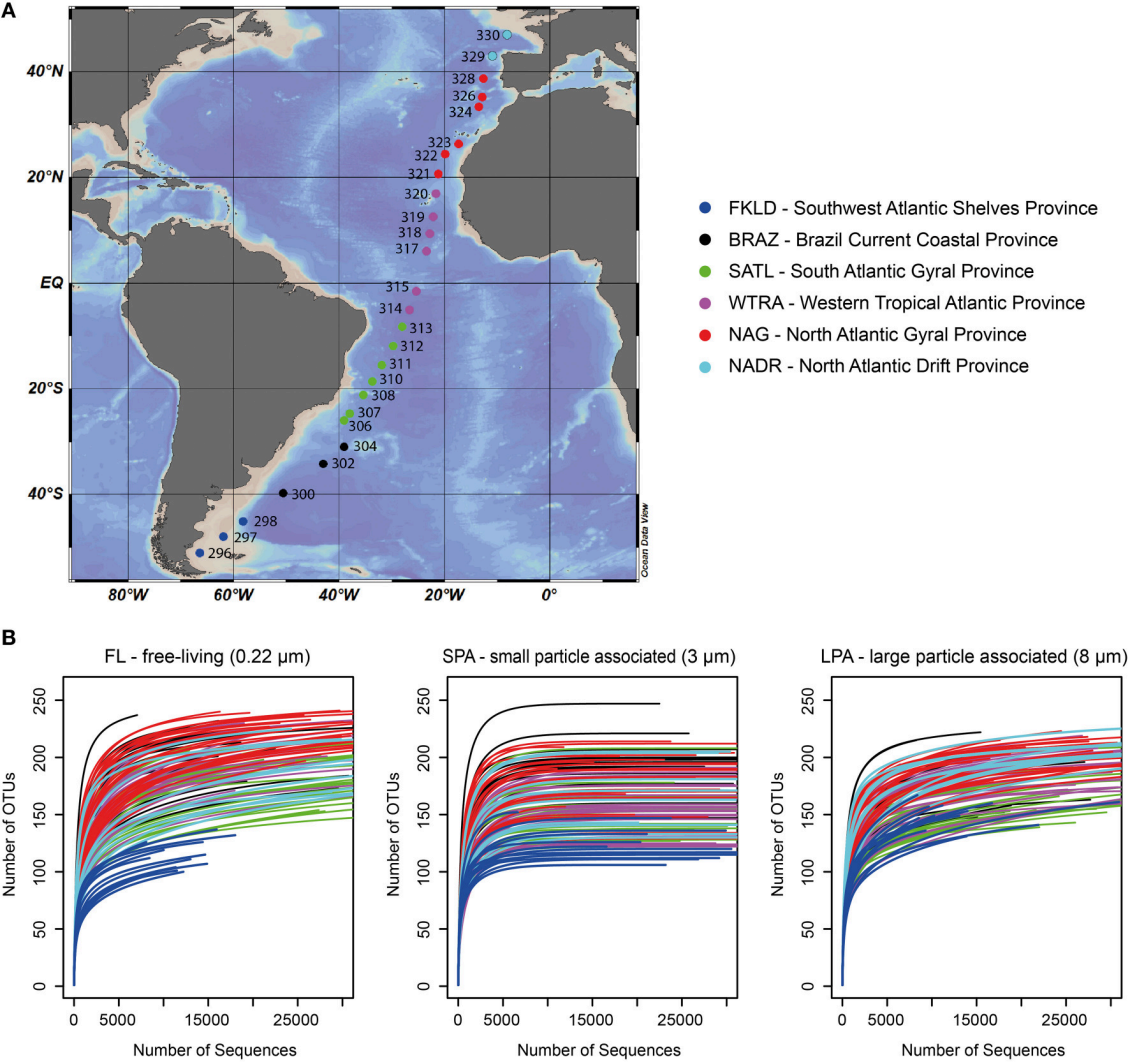
**Sampling sites and rarefaction analysis. (A)** Sampling sites across the Atlantic Ocean; for each station the original sampling ID is reported. **(B)** Rarefaction analysis for the three size fractions of bacterioplankton. The number of sequences per sample was limited to 30,000 sequences, the original raw data can be found in Table S3. For both panels color code distinguishes the six oceanographic provinces.

Samples were collected during the cruise ANT-28/5 (10 April–15 May 2012 with RV Polarstern) at 27 stations across a latitudinal transect in the Atlantic Ocean (51°S–47°N) (Figure 1A). At all stations, samples were consistently collected from five depths of the epipelagic zone: 20, 40, 60, 100, and 200 m; seven samples were taken ±10 m from the designated depths and additional eight samples were taken from intermediate depths (Table S1). To be able to analyze those samples together with the others, all samples were grouped according to five depth layers: 20, 40, 50–80, 85–120, and 140–200 m (Table S1). Water samples were fractionated using serial filtration as described (Milici et al., 2016b). Three size fractions of the plankton were therefore obtained: FL (free-living) for bacteria collected on the 0.22 μm membranes, SPA (small particle associated) for bacteria collected on the 3 μm membranes, and LPA (large particle associated) for bacteria collected on the 8 μm membranes.

DNA extraction and Illumina sequencing were performed as described (Milici et al., 2016b) and the raw data can be accessed at the ENA database (European Nucleotide Archive) BioProject ID: PRJEB11493.

### Taxonomic classification

Taxonomic classification was assigned using SINA aligner (version 1.2.11) (Pruesse et al., 2012) employing the reference database SILVA (119 NR) (Pruesse et al., 2007). The OTUs were aligned and classified against a maximum of 100 sequences that had a minimum of 97% similarity with the query sequence, using the lowest common ancestor method (LCA). The retrieved results were sorted for further analysis and processed in the following way: all the OTUs assigned to archaea or “unclassified” were excluded from the dataset and not considered further, since *in silico* analysis of the primer coverage revealed that only 18.2% of the archaeal sequences present in the SILVA database (123 NR) matched the primers used here. Sequences assigned to “chloroplast” were excluded from the bacterial table and analyzed separately with the PhytoREF database (Decelle et al., 2015) with BLAST version 2.2.28 (Camacho et al., 2009). All reads that had a similarity below 95% to known 16S sequences were excluded from the dataset. Results of the SINA classification were checked with BLAST (Camacho et al., 2009) version 2.2.28 against the SILVA 119 NR database (Pruesse et al., 2007). Some reads could be assigned only at the domain or phylum level with SINA (Table S2). For those ambiguous sequences a manual curated taxonomical assignment was performed (Figure S1). We first performed BLAST for the sequences against the SILVA database in order to understand whether the mis-classification was due to the absence of a reference or to misclassified sequences in the database. We found sequences that were assigned to two contrasting phylogenetic lineages of the domain bacteria (e.g., OTU_2_FL had 100% similarity both with a *Prochlorococcus* and OM1 clade sequence). Based on these results we downloaded a reference dataset constructed according to the BLAST results employing the SILVA database. We constructed alignments with MUSCLE and built phylogenetic trees with MEGA 6 using Neighbor-Joining method (Tamura et al., 2013).

Because of the difference in gene copy number across the different algae and bacterial lineages, read counts of 16S rRNA genes cannot be used to infer cell abundance. Here, we therefore report relative comparisons only using the term “relative abundance.” The taxonomic information and relative abundances of the OTUs identified in this study are reported in Table S3.

### Statistical analysis and network analysis

Statistical analyses were performed with PRIMER (v.7.0.6, PRIMER-E, Plymouth Marine Laboratory, Plymouth, UK; Clarke and Gorley, 2015), with the add on PERMANOVA+ (v. 1.0.6 PRIMER-E, Plymouth Marine Laboratory, Plymouth, UK; Anderson et al., 2006) and the statistical program R (https://www.r-project.org/, v. 3.0.1) with the library vegan: Community Ecology Package (v. 2.0-8). Rarefaction analysis was performed in R with library vegan, in order to assess if the sequencing effort was sufficient to investigate bacterial communities (Figure 1B). To infer correlations between OTUs and OTUs and environmental parameters, we first removed OTUs with a relative abundance below 0.1% of the total number of reads. We also removed all samples that had <7500 reads. Rare OTUs and samples with low sequencing depth might cause artifacts in the network analysis (Berry and Widder, 2014; Ju et al., 2014). Subsequently sample size for each of the three size fraction of the plankton was rarefied to 8,709, 10,125, and 7,780 sequences per sample in FL, SPA, and LPA respectively. The cumulative species dominance is shown in Figure S2.

Co-occurrences were then calculated with SparCC (Friedman and Alm, 2012) for each size fraction of the plankton and depth layer (20, 40, 50–80, 85–120, and 140–200 m) separately. Ten iterations were used to estimate the median correlation of each pairwise and the statistical significance of the correlations was calculated by bootstrapping with 500 iterations. Correction for multiple-testing of the *P* values was performed in R according to Benjamini-Hochberg method. Correlations were then sorted for statistical significance (*p* < 0.05) and *R* > ±0.6.

In order to account for the effect of indirect taxon edges driven by environmental parameters, methods from Lima-Mendez et al. (2015) were applied. For each taxon-environment union network, node triplets consisting of two taxa and one environmental parameter were identified in R. For each triplet, interaction information II was computed as II = CI(X, Y|Z) − I(X, Y), where CI is the conditional mutual information between taxa X and Y given environmental parameter Z, and I is the mutual information between X and Y. CI and I were estimated by using minet (Meyer et al., 2008). Taxon edges in environmental triplets were considered indirect when II < 0 and within the 0.05 quantile of the random II distribution obtained by shuffling environmental vectors (500 iterations). Furthermore, we checked if the triplets selected from the mutual information analysis were consistent in terms of sign patterns, as in Lima-Mendez et al. (2015). The original networks are reported in Table S4, and, the number of removed correlations is reported in Table S5.

To investigate associations between bacterial communities and photosynthetic micro-eukaryotes retrieved from the 8 μm filter, correlations were inferred with SparCC (Friedman and Alm, 2012). For this analysis, samples belonging to the last depth layer (140–200 m) as well as those with <1500 16S rRNA chloroplast sequences were excluded. Correction for multiple-testing of the *P* values was performed in R according to Benjamini-Hochberg method. Correlations were sorted for statistical significance (*p* < 0.05) and *R* > ±0.6. Networks were explored with Cytoscape, v 3.1.1 (Shannon et al., 2003). Topological parameters were calculated in Cytoscape with the tool network analyzer (Assenov et al., 2008).

## Results

### Overview of sequencing results and primers coverage

A total of 13.6 million reads were generated and roughly 11.2 million reads were affiliated to bacteria with a total of 259 OTUs for the FL (free-living bacteria, 0.22–3 μm), 269 OTUs for the SPA (small particle associated bacteria, 3–8 μm), and 236 OTUs for the LPA (large particle associated bacteria >8 μm) (Table S3). The average number of bacterial sequences per sample was similar for the three different communities: FL 27,552 ± 12,662, SPA 33,328 ± 10,586, and LPA 26,504 ± 12,446 (Table S3), despite the presence of photosynthetic eukaryotes especially in SPA and LPA. Rarefaction analysis showed that the vast majority of samples approached the plateau indicating a good sampling effort (Figure 1B). *In silico* test of primers coverage (F807 and R1050) (Bohorquez et al., 2012; Klindworth et al., 2013) showed a good coverage (86.4%) of bacterial lineages included in the SILVA database (version 123, NR). Taxonomic classification of the bacterial sequences showed that the majority of the OTUs (59–69%) could be affiliated at the order level, between 35 and 42% of OTUs could be affiliated to a family, and 16–24% were assigned to a genus (Table S6).

Because the primers used were universal for the prokaryotic small subunit RNA gene, they inevitably amplified also the plastidial 16S rRNA gene of eukaryotic microalgae. They were not found in the FL community (confirming the efficiency of the filtration), but amounted to ~6% in the SPA community and were termed SP (small phytoplankton) and ~21% in the LPA community and were termed LP (large phytoplankton). Seven different classes of microalgae could be identified (Table S3). An average of 1,094 ± 1,189 sequences for SP and 7,345 ± 10,504 for LP were generated. *In silico* analysis of the primer coverage of the marine photosynthetic eukaryotes (terrestrial lineages were excluded) included in the PhytoREF (Decelle et al., 2015) database revealed that primer sequences perfectly matched (no mismatch) 68% of the reference sequences (Table S7) and had one mismatch with 88% of the reference sequences (data not shown). Primers coverage differed for the various taxa; e.g., it was high for diatoms (92%) but low for haptophytes (8%) (Table S7). Although abundant, photosynthetic dinoflagellates are also under-represented in the reference database PhytoREF because of their specific plastidial genome organization (Decelle et al., 2015). These biases have to be taken into account when interpreting the data.

### Taxonomic composition of the bacterioplankton

**Figure 2:**
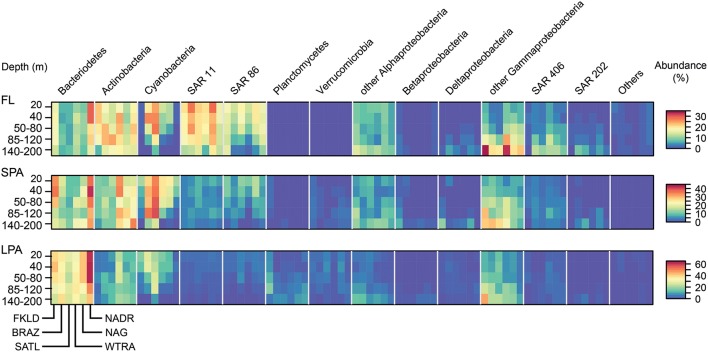
**Relative abundance of marine taxonomic groups of bacteria for the six oceanographic provinces across the whole photic zone and for three different size fractions**. The relative abundances of the main taxonomic groups (expressed in percent of total) were averaged for each province for all the five depth layers. OTUs were combined at the phylum level for most of the taxonomic groups, only Proteobacteria were grouped at the class level as same in all other figures. Important marine clades are shown separately because of their strong contribution to the bacterial communities. The remaining groups or unclassified bacterial OTUs are shown as “Others.” The heat map for each taxonomic group shows the five depth layers on the y-axis from the top to the bottom, and the six oceanographic provinces on the x-axis from South to North. Each taxonomic group is therefore composed of a block consisting of six times five rectangles, encompassing the provinces and depth layers, respectively. The three size fractions of the bacterioplankton (FL, SPA, LPA) are shown in three separate panels from top to bottom.

The relative abundance of the major marine groups of bacteria in the five depth layers of the epipelagic zone across the six oceanographic provinces covered in the cruise showed taxon specific distribution patterns according to filter size, province, and depth (Figure 2). Typical free living bacteria (clades SAR11, SAR86, SAR406, and SAR202) dominated the FL size fraction. By contrast, the LPA size fraction was dominated by Bacteroidetes, Actinobacteria, and Gammaproteobacteria other than SAR86. Planctomycetes and Verrucomicrobia were, absent in the FL community but found, in the SPA and LPA communities. Alphaproteobacteria other than SAR11 were found in both the FL and SPA size fraction at similar abundance, but represented a minor percentage in the LPA fraction.

Each province represented a unique environment in which specific taxonomic groups were enriched, depleted, or absent, with some general patterns that were conserved across the three size classes (FL, SPA, and LPA). Actinobacteria showed a transition from the South to the North of the transect. While in the FKLD and the BRAZ provinces they were enriched in the FL community, they became more abundant in the SPA size fraction from the SATL province onward. Bacteroidetes were more abundant in the two marginal provinces, where they reached the highest relative abundances. Cyanobacteria instead increased toward the equator with a peak in the SATL province, while Actinobacteria were more abundant in the WTRA province. SAR11 accounted for almost 30% of the total community in the NAG province.

Depth-related abundance patterns were also observed for most of the marine groups. For example, Cyanobacteria were depleted below 85 m except in the SPA community. SAR11 and SAR86 were more abundant above 120 m, while SAR406 and SAR202 were more abundant below 85 m. Gammaproteobacteria other than SAR86 accounted for more than 30% of all reads in the FKLD and WTRA province at 140–200 m.

**Figure 3:**
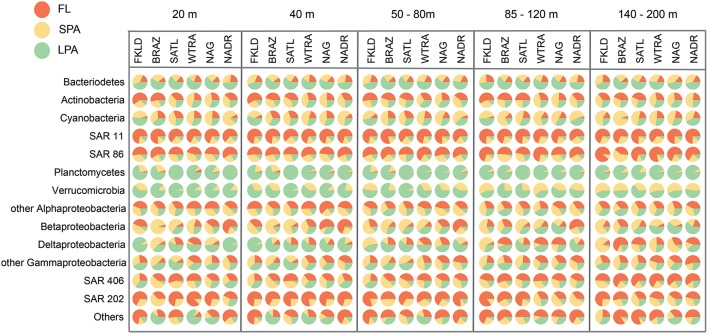
**Predominance of taxa in the different size classes of the bacterioplankton communities**. Each circle represents the average number of reads for the respective taxon, depth, and oceanographic province which was set to 100%. The percentage of reads from the three size fractions is shown by the area of the pie segments.

Figure 3 shows the relative proportion of reads on the three types of filters analyzed here for the same phylogenetic groups. More than half of all Bacteroidetes reads were found in the LPA community in all depths and provinces. Actinobacteria appeared to be heterogeneous, with more than 50% of reads in the FL community of the FKLD, BRAZ, and SATL province, but relatively even distribution between all three filter sizes in the other three provinces. About 75% of all SAR11 reads were found in the FL community; the remaining reads were mostly found in the SPA and some in the LPA community. Although SAR11 is a typical planktonic organism, it was previously detected in relatively high abundance in the particle attached fraction (Mohit et al., 2014). It is unclear if this is an artifact of the filtration procedure or a real biological finding, e.g., caused by mixotrophic eukaryotes, including microalga cells ingesting SAR11 (Hartmann et al., 2013).

SAR86, SAR406, and SAR202 showed a similar pattern, however the fraction of particle associated bacteria was higher for those clades. By contrast, nearly all of Planctomycetes and Verrumicrobia were found in the LPA or SPA size fraction. These groups are known for their sessile lifestyle (Pizzetti et al., 2011; Freitas et al., 2012). Reads from Alphaproteobacteria other than SAR11 were found on all filter sizes with varying percentage, in accordance with the different lifestyles and abundance patterns of globally distributed uncultivated clades (Voget et al., 2015) and algae-associated copiotrophs (Wagner-Döbler et al., 2010). The same holds true for Beta, Delta- and Gammaproteobacteria (other than SAR86) which are comprised of physiologically diverse species.

**Figure 4:**
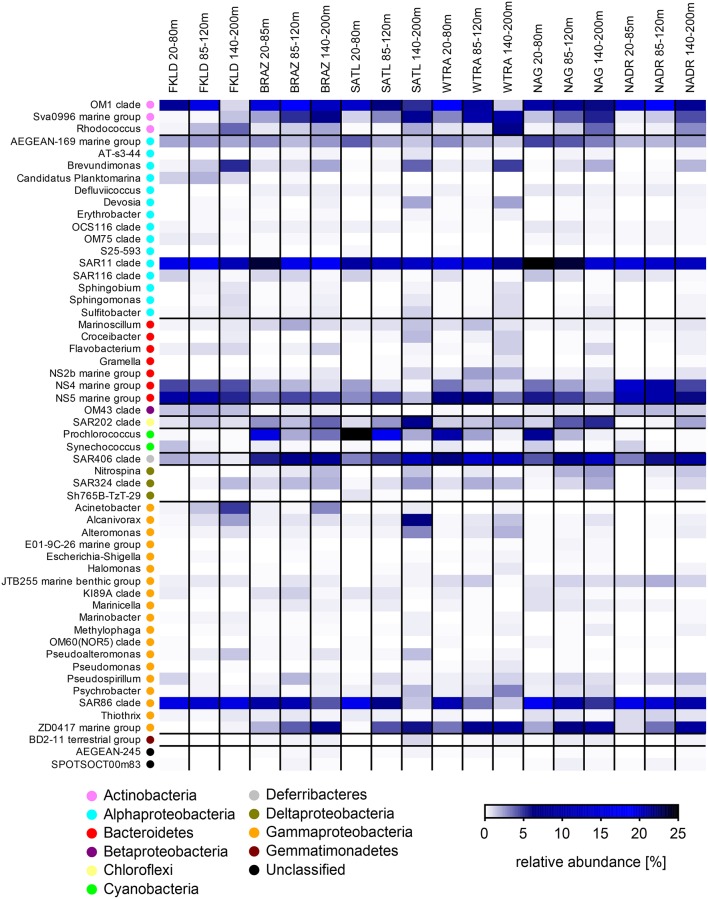
**Relative abundance of free-living (FL) marine bacterial clades and genera for the six oceanographic provinces across the whole photic zone:** Relative abundance of OTUs classified at the genus and clade level was averaged for the six oceanographic provinces in three depth layers (20–80, 85–120, and 140–200 m). Each line of the heat map represents a taxon, and its higher taxonomic rank (phylum or class) is indicated by the color key.

We then investigated relative abundance patterns at the level of clade or, if possible, genus, across depths, and provinces on the three filters (Figure 4, Figures S3, S4). Here, the differences in community composition along the oceanographic provinces and water column were even more striking. For example, *Planktomarina* sp. a Rhodobacteraceae (Giebel et al., 2013) was found almost exclusively in the FKLD province in the FL community. Acinetobacter was more abundant in the FKLD province and still found in the BRAZ province but almost absent throughout the rest of the transect. *Thalassospira* sp. was only found in the FKLD province and *Vibrio* sp. was more abundant in the WTRA province. The groups NS10 and NS7 were mainly represented in the FKLD and NADR provinces. Many more examples can be retrieved from the data, supporting the provincialism of bacterioplankton communities despite the absence of dispersal limitation across provinces (Milici et al., 2016a). The same is true for the clear depth related distribution of most microbial taxa. *Alcanivorax* sp., a known hydrocarbon degrading bacterium (Kostka et al., 2011), was found at high densities in the deeper part of the photic zone (140–200 m), accounting for up to 25% of all reads in the SATL and FKLD provinces. Several other taxonomic groups showed a gradual increase in relative abundance toward the deeper layers of the photic zone, e.g., *Rhodococcus* sp., *Brevudimonas* sp., and ZD0417, as well as most of the Gammaproteobacteria.

Beside regional patterns driven by provincialism and bathymetric stratification also widespread and abundant members of the community were observed. SAR11 and SAR86 were the most abundant taxa in the FL community in almost all provinces and depths. Interestingly, the relative abundance of the OM1 clade (Candidatus *Actinomarina minuta*; Ghai et al., 2013) was extremely high (~20%) throughout the whole transect. Clade AEGAN169 was also ubiquitous, but had a low abundance.

For the particles associated communities the shift in the taxonomic composition was even more pronounced at the genus and clade level. We observed several examples of specific lineages which were exclusively found in the SPA and LPA community like the NS7, NS9, and NS10 lineages of the Bacteroidetes. *Dinoroseobacter shibae*, a roseobacter which has been isolated from dinoflagellate cultures (Wagner-Döbler et al., 2010), was found in the SPA community of the FKLD province. Within the Deltaproteobacteria, some lineages were found exclusively on the particles like the OM27 and GR-WP33-58 clades/groups. Similarly, lineages within the Verrucomicrobia (e.g., Arctic97B-4 marine group and *Roseibacillus* sp.) and the Planctomycetes (e.g., *Blastopirellula* sp. and *Planctomyces* sp.) were found exclusively on particles. Many other examples of taxonomic groups specifically enriched on one of the size fractions of the bacterioplankton can be retrieved from Figures S3, S4. Those results suggest that particles select for specific microbial communities and require genomic traits present in various microbial lineages.

### Distribution and taxonomic composition of photosynthetic eukaryotes

**Figure 5:**
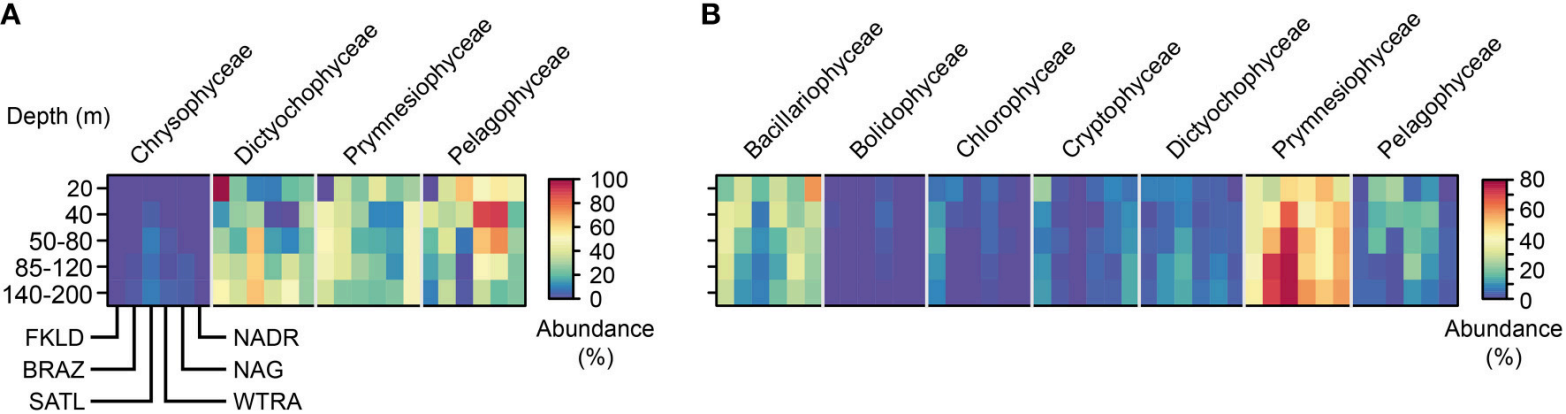
**Taxonomic composition of micro-eukaryote communities across the whole photic zone**. Relative abundances for the main taxonomic groups were averaged for the five depth layers in each province. In panel **(A)** the micro-eukaryotes collected on the 3 μm filters (SP) are shown, while in panel **(B)** the micro-eukaryotes collected on the 8 μm filters (LP) are reported. OTUs were grouped at the taxonomic level of class. Each single heat map shows the five depth layers on the y-axis from the top to the bottom, and the six oceanographic provinces from South to North on the x-axis. Each taxonomic group is therefore composed of a block consisting of six times five rectangles, encompassing the provinces and depth layers, respectively.

The sequences from the plastidial 16S rRNA genes of photosynthetic eukaryotes obtained in this study were analyzed using the reference database PhytoREF (Decelle et al., 2015) and have unambiguously been assigned with high percentage similarity (from 95 to 100%) to reference sequences (Table S3). In the LP communities (>8 μm) seven classes of eukaryotic microalgae were identified, from which Bacillariophyceae (diatoms) and Prymnesiophyceae (haptophytes) were numerically the most prevalent (Figure 5) and showed a clear biogeographical pattern. Bacillariophyceae were more represented in the marginal provinces (FKLD and NADR) with strong depletion in relative abundances in the more oligotrophic SATL province. By contrast, Prymnesiophyceae were the most abundant group from the BRAZ to the NAG province with peaks in the BRAZ and SATL province. Pelagophyceae and Dictyophyceae were very rare in the FKLD and NADR provinces and more abundant in the BRAZ and SATL province. Cryptophyceae showed an opposite pattern with an enrichment in the FKLD and NADR provinces and were almost absent throughout the rest of the transect. Moreover, photosynthetic eukaryotes showed patterns related to depth: Prymnesiophyceae for instance increased in the deeper part of the photic zone, especially in the BRAZ and SATL provinces, in contrast to most other algae. The Dictyochophyceae were also enriched in the 140–200 m depth layer of the BRAZ and SATL provinces.

In the SPA community four classes of eukaryotic microalgae were identified and displayed a complex biogeographical pattern (Figure 5). Chrysophyceae and Dictyophyceae showed higher abundances in the central provinces (SATL, WTRA, and NAG). Pelagophyceae were depleted, while Dictyochophyceae were more abundant in the deeper part of the photic zone. Remarkably, we detected *Braarudosphaera bigelowii* (99% identity with query sequence) a coccolithophore, known for its symbiotic relationship with a cyanobacterium (Thompson et al., 2012). However, we did not find any sequence which was affiliated to the lineage of cyanobacteria UCYN-A.

### Numerical description of co-occurrence networks

**Figure 6:**
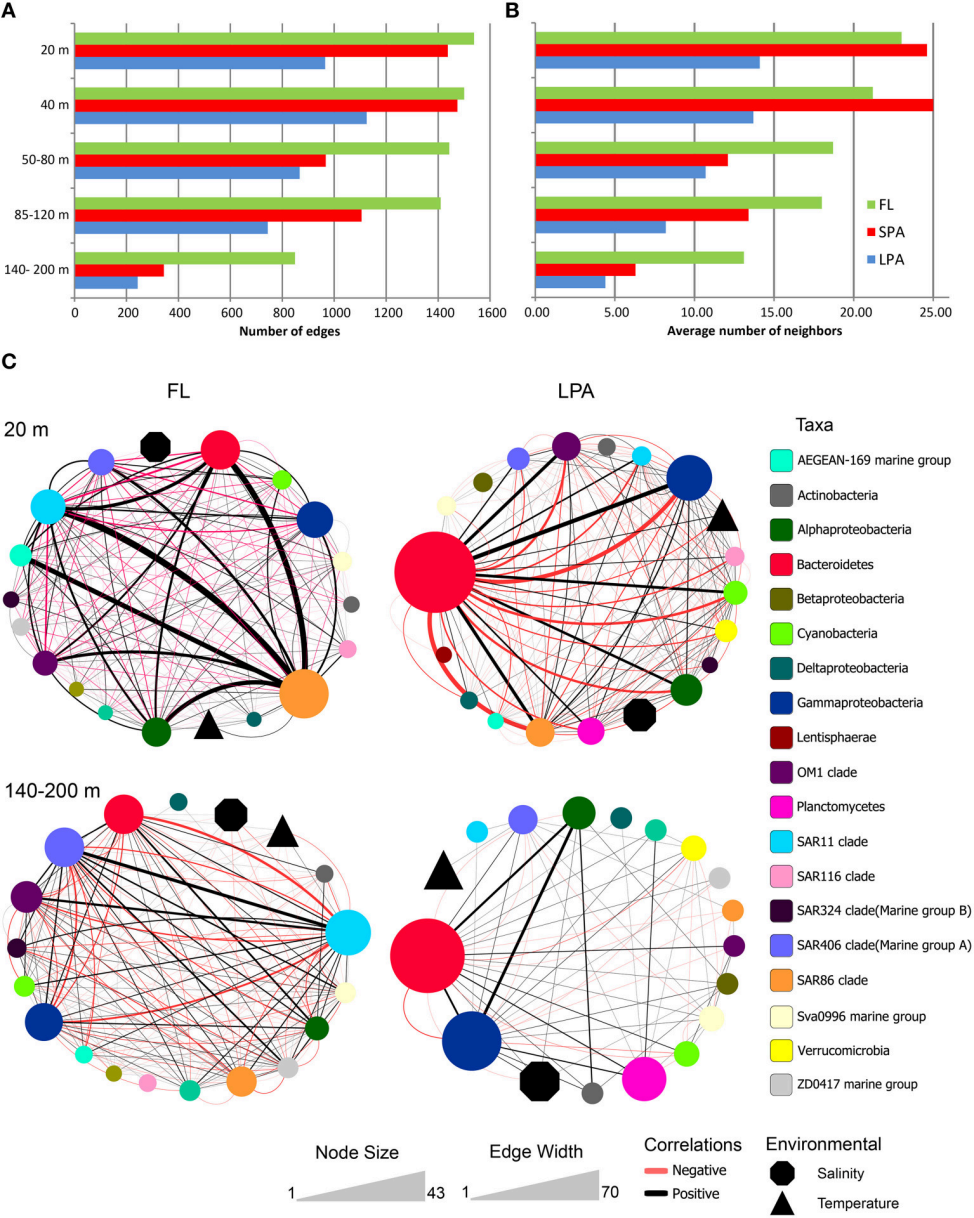
**Bacterioplankton co-occurrence networks**. Network analysis was performed at the OTU level for each depth layer and size fraction of the plankton. Selected topological parameters of the networks are plotted: **(A)** Number of edges in each network and **(B)** average number of neighbors per node. See Tables S8–S10 for the full set of network parameters. **(C)** The four most extreme networks are displayed: The 20 and 140–200 m depth layer (top to down) of the FL and LPA communities (left to right), respectively. In order to better display co-occurrence patterns OTUs were grouped at different taxonomic level (known marine clades as well as classes and phyla). The size of each oval (node) indicates the number of OTUs belonging to that taxonomic group, while the size of environmental parameters (temperature and salinity) was arbitrary defined. Lines connecting two nodes represent a group of strong (*R* > 0.6) and significant correlations (*p* < 0.05). Black lines indicate a positive correlation while red lines indicate a negative correlation. The width of each line exhibit the number of correlations occurring among the two connected nodes.

From the 15 networks generated we observed that the number of edges and the average number of neighbors were higher in the FL community and decreased in the SPA and LPA communities, as shown in Figure 6 and reported in Tables S8–S10. For instance we observed that in the 20 m depth layer the number of edges was 1538 in the FL community and decreased to 965 in the LPA size fraction despite the comparable number of nodes (134 and 137, respectively for the FL and LPA). These results are reflected in the average number of neighbors per node of the two networks (FL and LPA 20 m depth layer) which were 23 and 14.1 respectively. Overall, those data suggest a higher number of co-occurrences in the FL community. This result was not affected by the number of negative correlations (see below) and in fact when the negative correlations were removed, it was observed that the FL community still had the highest number of neighbors per node throughout the whole water column (Table S11).

Furthermore, we observed that the number of edges of the networks within each size class of the plankton (FL, SPA, and LPA) decreased with depth, reaching the minimum in the 140–200 m depth layer. In the FL fraction 1538 edges were retrieved in the 20 m depth layer while only 849 were found in the 140–200 m depth layer (Figure 6). This shift was even more dramatic in the SPA and LPA fractions, where we found 1437 and 965 edges for the 20 m depth layer, while only 344 and 243 were retrieved from the 140–200 m depth layer, respectively. Overall in the last part of the photic zone consistently 50–75% fewer correlations were found. Analysis of the other topological parameters of the networks (Tables S8–S10) supported those findings and showed a reduction in the complexity of the networks from the upper (20 m) toward the lower epipelagic zone (140–200 m).

Overall, those results show that particle associated bacteria, although living in close physical contact, tended to establish a much lower number of co-occurrences compared to the free living planktonic bacteria.

### Distribution of negative and positive correlations in the co-occurrence networks

**Figure 7:**
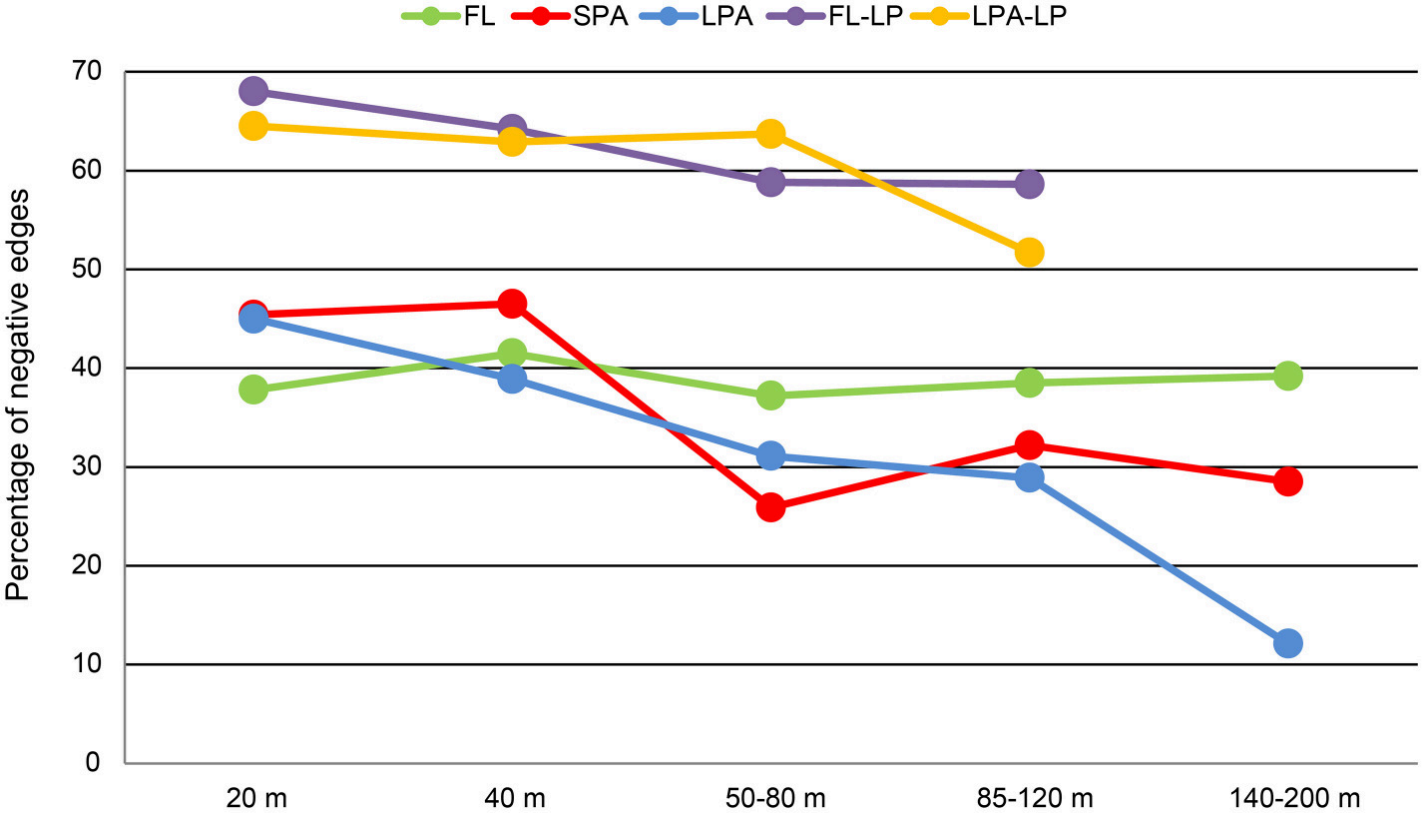
**Bathymetric distribution of negative correlations**. For each of the networks generated (15 bacteria-bacteria networks and 8 bacteria-micro-eukaryotes networks) the relative amount of negative correlations is plotted against depth. Different colors distinguish the networks: FL, free-living bacteria; SPA, small particle associated bacteria; LPA, large particle associated bacteria; FL-LP, free-living bacteria and micro-eukaryotes retrieved from the 8 μm membranes; and LPA-LP, large particle associated bacteria and micro-eukaryotes retrieved from the 8 μm membranes.

We investigated the distribution of the negative and positive correlations along the water column and in different size classes of the plankton (Figure 7). Our analysis revealed that the number of negative co-occurrences was higher in the FL fraction of the plankton (Tables S12–S14). These accounted for roughly 40% of the total edges and did not follow any bathymetrical pattern. Interestingly, these results were in contrast to the particle associated fraction of the plankton. In both SPA and LPA communities we found that the number of negative associations was higher in the 20 and 40 m depth layers where they amounted for 40–47% of the total. However, they were already reduced in the 60 m depth layer (26–31%) and reached their minimum in the 140–200 m depth layer of the LPA fraction where they accounted for 12% of the total number of co-occurrences (Figure 7).

### Taxonomic structure of co-occurrence networks

**Figure 8:**
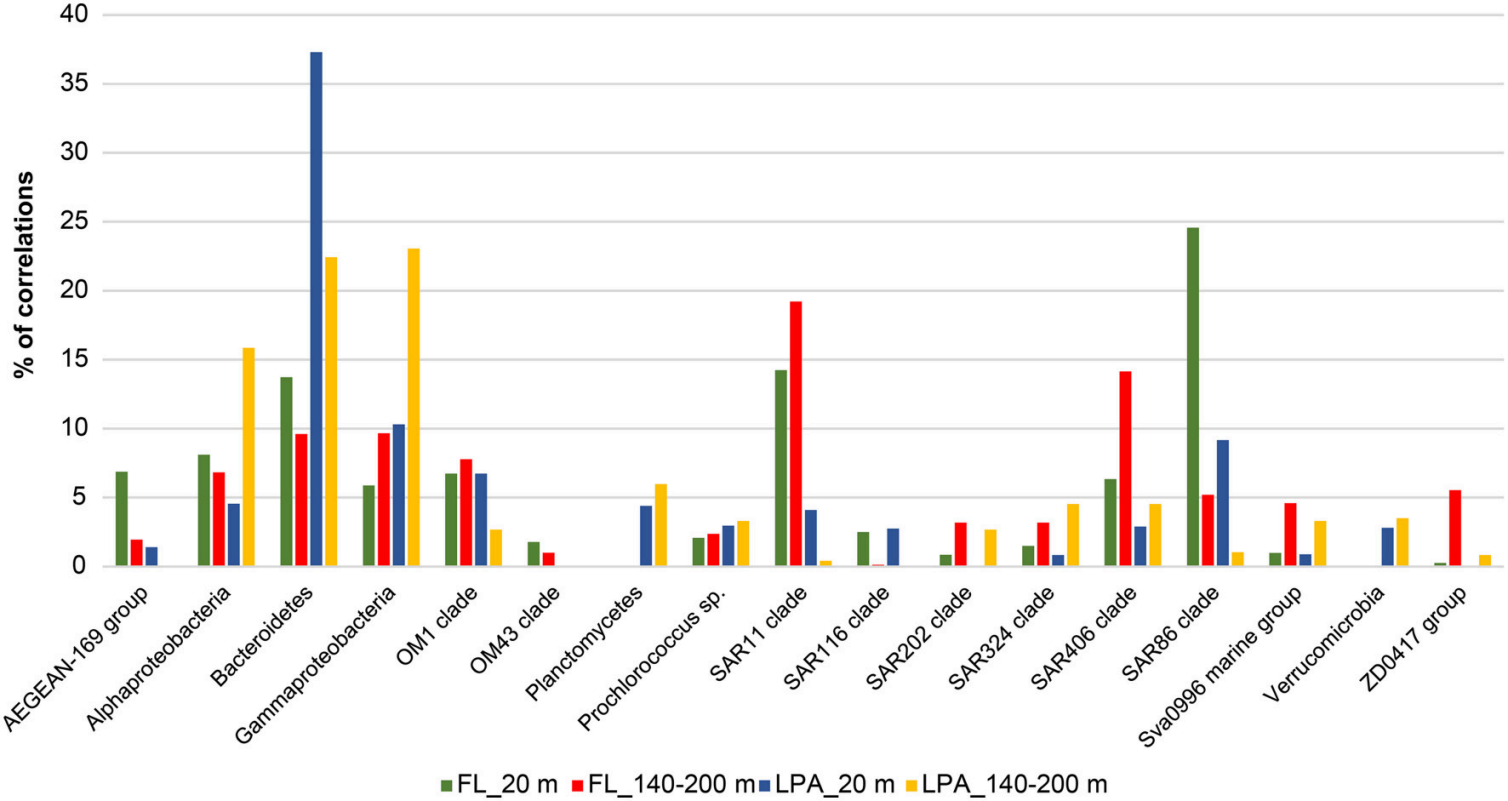
**Relative contribution to the total number of correlations for different taxonomic groups for the depth stratified networks:** Network analysis was performed at the OTU level for each depth layer and size fraction of the plankton as described in Materials and Methods. Nodes belonging to the same taxonomic group (known important marine clades as well as classes and phyla of the bacterioplankton) were grouped together and the relative amount of correlations was calculated and expressed as percentage of the total number of correlations of the network. Four networks are here displayed (FL_20, FL_140-200, LPA_20, and LPA140_200 m) with different colors.

We then investigated the taxonomic structure of the networks on the level of OTU (Figure 6). Co-occurrence patterns demonstrated a high degree of association across different phyla and classes, as well as within them, and those association patterns changed across the three size fractions of the plankton and along the water column. In the 20 m depth layer of the FL network, we found that two clades alone (SAR11 and SAR86) harbored more than 40% of the total correlations of the network (Figure 8). Moreover, other oligotrophic taxa like AEGEAN-169, OM1, and SAR406 contributed between 6 and 7% to the total correlation number. In the FL 140–200 depth layer network, we observed a shift in the dominance (in terms of percentage of correlations) of some taxonomic group when compared to the upper layer of the epipelagic zone. The SAR406 clade in fact doubled the amount of total co-occurrences reaching up 15% of the total. Interestingly, while the SAR86 clade showed a dramatic reduction of the number of association (only 5% of the total in the 140–200 m depth layer) the clade SAR11 showed an increased amount of correlations that accounted for 20% of the total network. Other taxa like the SAR 202, SAR 324, OM1, Sva0996, and the ZD0417 contributed between 3 and 8% of the total correlations. In the LPA size class of the plankton Bacteroidetes alone had roughly one third (37%) of the total correlations of the network with Planctomycetes and Verrucomicrobia contributing to 4 and 3% of the network co-occurrences and Gammaproteobacteria accounting for about 10%. In the LPA 140–200 m network, the amount of co-occurrences attributed to Bacteroidetes decreased to 22%, while those derived from the Gammaproteobacteria increased to 23% of the network. Alphaproteobacteria were found to have roughly 15% of the total correlations of the network, with Planctomycetes, Verrucomicrobia, SAR324, SAR406, SAR 202, and Sva0996 contributing between 2 and 5% of the total co-occurrences. Overall it emerges that the co-occurrence patterns strongly changed along the water column and between different size fractions of the plankton. The FL networks were dominated by oligotrophic clades like the SAR11, SAR86, and SAR406. Those were only marginally retrieved in the LPA size class and probably reflect trophic relationship within planktonic communities i.e., protist collected on the 8 μm membrane were feeding on the most dominant free-living bacteria (Hartmann et al., 2012, 2013). The LPA community was instead dominated by groups of microorganisms like Bacteroidetes, Gammaproteobacteria as well as the Alphaproteobacteria not affiliated to known streamlined marine lineages (Swan et al., 2013).

### Associations between eukaryotic microalgae and bacteria

**Figure 9:**
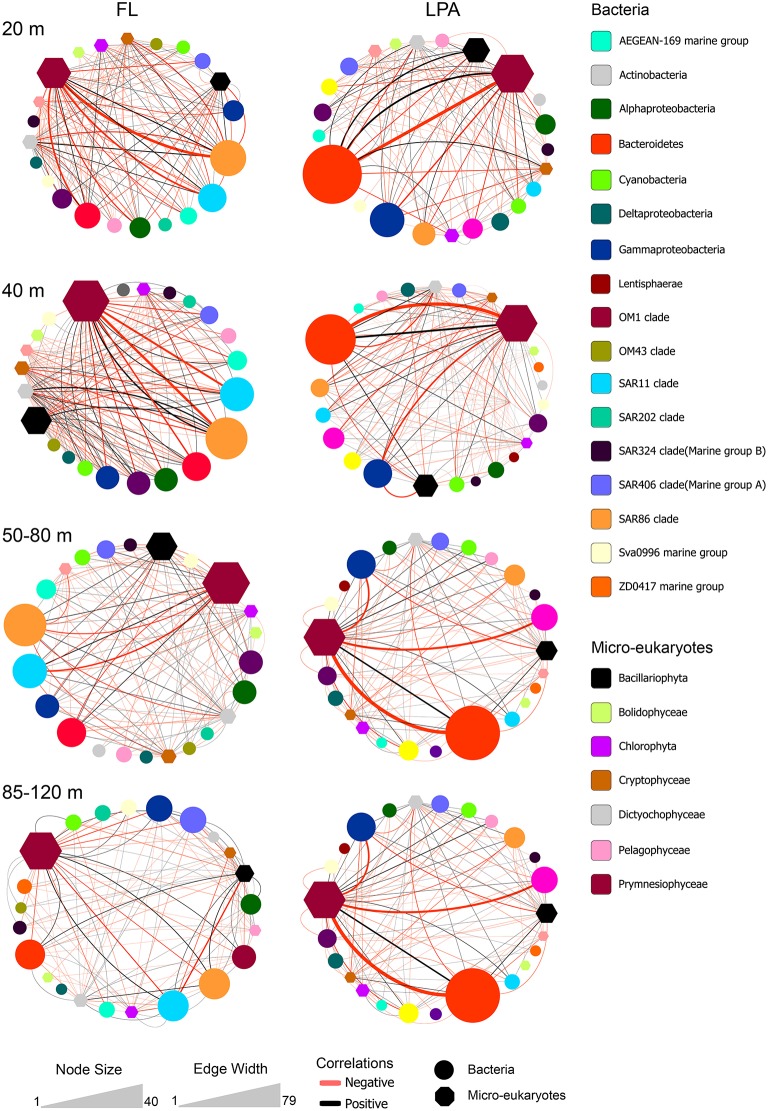
**Bacterial co-occurrences with micro-eukaryotes**. Correlations between micro-eukaryotes and bacteria were calculated at the OTU level for four different depth layers: 20, 40, 50–80, and 85–120 m (top to bottom) between the bacterial OTUs of the FL (left) and LPA (right) communities with the micro-eukaryotes retrieved on the 8 μm filters (LP). Color code indicates the taxonomic assignment of each node while the shape distinguishes between bacteria and micro-eukaryotes (oval or hexagon). The size of each node reflects the number of OTUs affiliated to each taxon. Co-occurrences between taxa are displayed by lines. The width of each line indicates the number of correlations occurring among the two connected nodes, while the color reflects positive (black) or negative (red) associations.

To investigate algae-bacteria co-occurrences, correlations were calculated between bacterial present OTUs in the FL and LPA communities with the micro-eukaryotes found on the 8 μm membranes. Analysis of the topological coefficients of the two networks (FL bacteria with micro-eukaryotes and LPA bacteria with micro-eukaryotes) revealed a reduction of the number of nodes and edges from the 20 m depth layer toward the 85–120 m. Moreover, the average number of neighbors decreased with depth while the characteristic path length increased (Tables S15, S16). Overall those analyses suggest a bathymetric stratification of the networks similar to what reported for the bacterial networks. In the FL community Alphaproteobacteria and Gammaproteobacteria, which includes the highly abundant SAR11 and SAR86 clades, dominated the network (Figure 9). Most of the correlations occurred with members of the Prymnesiophyceae and Dictyophyta. In the LPA community the co-occurrence networks were more complex showing additional associations with Bacteroidetes, Verrucomicrobia, and Planctomycetes. Furthermore, we found that most of the edges (~60%) analyzed here were negative (Figure 7). These were higher in the upper layers (20 m), where amounted for 65–68% of the total, and slightly decreased toward the 85–120 m depth layer (54–58%) (Table S17 and Figure 7).

From our analysis it emerged that the association patterns between phytoplankton and bacteria were not strictly taxonomically constrained, i.e., the same bacterium was not found to be always associated with the same algal OTUs. This, in our opinion, suggests that the associations between phytoplankton and bacteria are rather opportunistic and might follow stochastic processes.

Most of the associations were negative which might indicate top-down processes and antagonistic relationships.

## Discussion

### Taxonomic composition of bacterioplankton communities

Here we characterized bacterial communities of a transect across the Atlantic Ocean with Illumina sequencing of the V5-V6 region of the 16S rRNA gene. Overall, our taxonomic investigation was in accordance with previous studies with respect to the relative abundances of many known marine groups (Rusch et al., 2007; Schattenhofer et al., 2009; Friedline et al., 2012; Morris R. M. et al., 2012; Sunagawa et al., 2015). Actinobacteria were found to be more abundant when compared to other studies (Rusch et al., 2007; Morris R. M. et al., 2012; Sunagawa et al., 2015). This finding was related to the high relative abundance of the recently characterized *Actinominuta temperata* (OM1 clade) (Ghai et al., 2013). This clade of the marine bacterioplankton was described as having one of the smallest cell sizes described so far in the ocean, with an estimated genome size <1 Mb (Ghai et al., 2013), and hence represents a typical oligotrophic organism with a streamlined genome (Giovannoni et al., 2014). The clade was found to be extremely abundant throughout the whole transect (~20%) highlighting its importance in the marine ecosystem together with the other abundant and widespread oligotrophic bacterial lineages like SAR11, SAR86, and *Prochlorococcus* sp. (Morris et al., 2002; Rusch et al., 2007; Dupont et al., 2012; Sunagawa et al., 2015).

The biogeography of most of the taxa identified here was related to three main parameters: province, depth layer, and filter size and will be discussed below.

#### Province

Six biogeochemical provinces according to Longhurst were covered (Longhurst, 1998). The concept of biogeochemical provinces was developed to predict algal blooms in the ocean. It is based on the dynamics of surface temperature, salinity, bathymetry and chlorophyll *a* concentration and has been refined ever since (Reygondeau et al., 2013). Thus, these provinces provide an ecologically tremendously useful framework for interpreting biogeographical data. The different provinces of the transect had different community composition, with enrichment and depletion of specific taxa, like the clade SAR11 which was more abundant in the FL community of the BRAZ and NAG provinces. Other examples of taxa with a province related distribution (provincialism) were observed, among those a remarkable example was Bacteroidetes. This group of bacteria is known to be associated to phytoplankton (Gómez-Pereira et al., 2012; Buchan et al., 2014); they are able to metabolize polysaccharides produced by the phytoplankton and dominate the community during algal blooms (Teeling et al., 2012; Wemheuer et al., 2014). Here we show that they comprised one of the most important phyla in the two marginal provinces of the transect: FKLD and NADR, which are highly productive areas of the Atlantic Ocean (Chust et al., 2013; De Monte et al., 2013).

#### Depth

We observed a shift in community composition along the water column, which mainly involved members of the Alphaproteobacteria and Gammaproteobacteria as well as the clades SAR202 and SAR406. The deeper layers of the photic zone might represent a transition environment between the deep ocean (>200 m) and the euphotic zone. Members of the SAR202 and SAR406 are extremely important in the mesopelagic and bathypelagic zones of all oceans (Salazar et al., 2015), and seem like many other Gammaproteobacteria to be adapted to light reduced environments. Those taxa were the most abundant across the complete transect in the 140–200 m depth layer. The pattern was conserved for all three filter sizes, indicating that the deeper layers of the photic zone (140–200 m) represent a unique environment. Despite the extensive sampling area that covered almost the entire Atlantic Ocean, those communities were remarkably similar. These findings corroborate the hypothesis (Aristegui et al., 2009) that systems not driven by light, e.g., the dark ocean, are more homogeneous than the photic zone with respect to their community composition and with respect to diversity patterns.

#### Filter size

Our taxonomic analysis showed that the three filters harbored different communities, with enrichment of Planctomycetes, Verrucomicrobia, Deltaproteobacteria, and Bactereoidetes on the particles and depletion of taxa like SAR11 and SAR86, in agreement with previous studies carried out in the Atlantic Ocean and other regions (DeLong et al., 1993; Crump et al., 1999; Ganesh et al., 2014; Mohit et al., 2014; Bižic-Ionescu et al., 2015; Fontanez et al., 2015; Salazar et al., 2015). However, these results concern only higher taxonomic ranks like phylum and class. When investigated at the clade and genus level, we observed that the three size fractions of the plankton analyzed here harbored different taxa for almost each of the phyla and classes identified. These data show that the particle associated lifestyle is a widespread feature of marine bacteria, and present among all phyla and classes of the bacterioplankton. However, some phyla like Planctomycetes and Verrucomicrobia seem to have evolved only a particle associated lifestyle, while in groups like the Alphaproteobacteria and Gammaproteobacteria both lifestyles are present. Those findings suggest, as recently reported for the deep ocean (Salazar et al., 2015), that the particle associated lifestyle is a conserved trait of the bacterioplankton also in the photic zone, and that more emphasis should be put in studying microbial communities associated to particles. However, it must be taken into account that those results are likely to be influenced by filtration biases (Padilla et al., 2015) as well as top-down processes like predation, i.e., free-living bacteria that were ingested by eukaryotes were retained on the bigger size fraction of the plankton. Those issues are hard to solve and in fact the free-living bacteria like SAR11, SAR86, and *Prochlorococcus* sp. have been found to be highly abundant on particles in previous studies (Crespo et al., 2013; Bižic-Ionescu et al., 2015; Rieck et al., 2015).

Bacterioplankton community composition is strongly influenced by seasonal processes (Gilbert et al., 2012; Bryant et al., 2015; Fuhrman et al., 2015). In our study, the sampling time was roughly 5 weeks and, therefore, our data do not address seasonality and are influenced by the time of the year sampled (April–May).

### Comparison of our networks with previously published planktonic networks

Previous analyses have investigated the network composition and structure of planktonic communities with particular emphasis on time series data. It is therefore worthwhile to compare the complexity of previous networks in terms of number of nodes and edges. In a recent depth stratified analysis of bacterioplankton communities (Cram et al., 2015b), it was shown that the total number of nodes per layer was between 57 (890 m depth) and 83 (150 m depth) and in total 537 nodes with 2301 edges were found. A time series study at the SPOT station revealed that the bacteria-bacteria network was constituted by 226 nodes and 837 edges (Chow et al., 2013). Peura and colleagues reported a total of 308 nodes and 1436 edges for the HOT station (Peura et al., 2015). In the *Tara* Oceans study several networks were constructed for the different size classes of the plankton as well as for the two depth layers (Lima-Mendez et al., 2015). For the bacterioplankton networks from the FL fraction, a total of 716 and 795 nodes were found for the surface samples (5 m) and the DCM (deep chlorophyll maximum), with respectively 1749 and 2838 edges. Thus, differences in the methods of network construction as well as sequencing method strongly affect the network structure and complexity (Faust and Raes, 2012; Faust et al., 2015a,b). However, from those data it emerges that in our study a higher number of correlations per node was retrieved in the FL community, also if compared to the *Tara* oceans study. This might reflect the narrower geographical range studied here and the different sequencing method used.

### Network analysis shows highly interconnected networks for free-living bacterioplankton

Analysis of the topological parameters of the three networks (FL, SPA, and LPA) revealed a higher number of correlations in the FL community while the particle fractions showed a reduced number of correlations. Those findings are to a certain extent illogical: why are particle associated bacteria establishing fewer co-occurrences than the free-living ones, in spite of their close physical neighborhood? According to the “lottery hypothesis” particles might be colonized by chance with a few phylotypes out of the huge diversity of bacteria with similar functional traits, as suggested for the *Ulva australis* holobiont (Sale, 1979; Burke et al., 2011a,b). Metagenomic studies reported that particle associated bacteria have on average a larger genome (Zeigler et al., 2012; Allen et al., 2013), with enrichment of genomic traits that mediate interactions, vitamins synthesis, and polysaccharide degradation (Smith et al., 2013; Ganesh et al., 2014; Satinsky et al., 2014; Simon et al., 2014) as well as genes related to competition (Ganesh et al., 2014), supporting the idea that lineages enriched on particles have a copiotrophic lifestyle rather than an oligotrophic one (Allen et al., 2013). The higher number of genes might represent an advantage in a nutrient rich environment (Giovannoni et al., 2014) allowing them to adapt quickly to available nutrients without the need to establish interactions with neighboring microorganisms. On the other hand, free-living bacteria often have a streamlined genome (Swan et al., 2013), which has been proposed as a competitive advantage in nutrient poor environments (Giovannoni et al., 2014). Many members of the bacterioplankton lack metabolic pathways for the synthesis of essential nutrients like vitamins (Giovannoni, 2012; Sañudo-Wilhelmy et al., 2014).

The most abundant bacterioplankton taxa, like SAR11, SAR86 and *Prochlorococcus* sp. have streamlined genomes nevertheless, they dominate bacterioplankton communities on a planetary scale (Rusch et al., 2007; Sunagawa et al., 2015). In this study, we observed that the candidatus *A. minuta* (OM1 clade), which has been described as the smallest marine bacterium, with a genome that might be smaller than 1 Mb (Ghai et al., 2013), is also extremely abundant. The authors proposed a photoheterotrophic lifestyle and further suggested association with cyanobacteria (Ghai et al., 2013). Here we showed that the clade was also abundant in regions were cyanobacteria were almost undetectable (FKLD) suggesting that its relative abundance does not depend upon the presence of cyanobacteria.

How can those microorganisms be so successful despite the absence of essential genes? Morris and colleagues postulated the “black queen hypothesis” (BQH) (Morris J. J. et al., 2012) stating that members of the microbial community can be classified as “helpers” and “beneficiaries.” Beneficiaries are abundant genome streamlined organisms that rely on helpers for essential metabolites or other “services” that allow their survival and ecological success; in turn, they provide the helpers with metabolites. Based on a systematic analysis of possible metabolic interactions in 800 different microbial communities from a wide range of environments (soil, aquatic, marine, and human gut) Zelezniak et al. (2015) concluded that microbial communities harbor highly interconnected groups of taxa that exchange metabolites like sugars and amino acids and co-occur in different habitats. The interdependencies among different microorganisms would thereby explain the inability of culturing marine bacteria. A recent paper which used a mixed culture approach (Garcia et al., 2015) based on metagenomics data suggested that Crenarcheota provided the cofactor B12 to the bacterial members of the community. Interestingly, a time series analysis of the SPOT station, during a time frame of roughly 3 years has shown that members of the SAR86, as well as of the SAR11 clades strongly co-occurred with Crenarcheota suggesting a possible interdependency since both clades (SAR11 and SAR86) are auxotrophic for cobalamin (Beman et al., 2011). A recent network analysis of freshwater and marine bacterioplankton accordingly found that the abundant taxa, which typically have streamlined genomes, tend to have a higher number of co-occurrences (Peura et al., 2015). A time series analysis of metatranscriptome data from two different marine environments established functional community networks which showed high inter-taxa correlations and coordination of several metabolic pathways which was taxonomy independent but rather time related indicating the existence of complex patterns of metabolic interdependency which are not only spatially constrained but also time dependent (Aylward et al., 2015). Genes related to amino acid metabolism, DNA processing, translation, transporters, and vitamin metabolism had the highest connectivity of all transcripts which suggests a tight regulation of those features within the community (Aylward et al., 2015). Taking all findings together, the higher abundance of bacteria with streamlined genomes (SAR11, SAR 86, OM1, and *Prochlorococcus* sp.) in the FL community might explain the larger number of correlations observed. Those bacteria are critically dependent on metabolites which might be provided by the surrounding members of the planktonic communities, suggesting that the BQH could be one of the mechanisms contributing to network topology in the FL fraction. Species living on the particles instead have large genomes that might allow them to be more independent.

### Depth stratified co-occurrences networks

We observed that the number of co-occurrences between bacterial OTUs decreased with depth and was lowest in the 140–200 m depth layer for all size fraction of the plankton, which, to the best of our knowledge, has not been observed before (Chow et al., 2013; Cram et al., 2015a,b). By contrast, in the *Tara* oceans study (Lima-Mendez et al., 2015) it was shown that bacterioplankton communities collected on 0.22 μm membranes had a much higher number of correlations in the DCM compared to surface samples (5 m). Overall, those studies suggest that the stratification of planktonic communities does not only involve a shift in the taxonomic composition (Eiler et al., 2011; Vergin et al., 2013; Sunagawa et al., 2015) and in the diversity (Bryant et al., 2015; Cram et al., 2015a; Sunagawa et al., 2015; Milici et al., 2016b) but also in the network structure and co-occurrence patterns. The link and intersection between planktonic networks from different depth layers can help us to understand the global carbon cycle (Guidi et al., 2016).

### Negative correlations are an important driver of the free-living fraction of the plankton

In our analysis, we found that roughly one third of the correlations retrieved in the FL community of the bacteriopalnkton were negative. This result indicates that mutual exclusion plays an important role in shaping community composition and could be caused by antagonistic associations or reflect niche partitioning (Chaffron et al., 2010; Hibbing et al., 2010; Freilich et al., 2011). Studies on time series data have reported a comparable amount of negative correlations, e.g., Chow and colleagues reported that negative associations accounted for ~30% at SPOT (Chow et al., 2014) and comparable results were found for lake bacterioplankton (Eiler et al., 2012). Co-occurrence analyses over large geographical distance have also reported a similar amount of negative correlations (~25%) (Lima-Mendez et al., 2015) although most of them occurred between eukaryotes and between bacteria and eukaryotes, suggesting almost no antagonistic processes among bacterioplankton. However, when only prokaryotic communities were analyzed, it emerged that negative co-occurrences amounted for roughly 11.5% of the surface (5 m) network and only 1.5% of the DCM network. Those values are significantly smaller than ours (~35%). It must be taken into account that in our study the sampling time was much shorter (5 weeks) compared to the 4 years of the *Tara* Oceans expedition. Co-occurrences are known to follow seasonal patterns (Fuhrman et al., 2015). Moreover, the 300 samples from the *Tara* Oceans study were derived from all oceans and all size fractions of the plankton (from viruses to mesoplankton), thus the study is based on a geographically much more heterogeneous sample set. Yet in spite of discrepancies in the relative abundance of negative correlations, it clearly emerges that they are commonly retrieved in the FL fraction of the bacterioplankton.

We also show that the number of negative correlations decreased in the particle associated fraction of the bacterioplankton (SPA and LPA) and in particular in the 140–200 m depth layer of the LPA community. Those findings might suggest that particle-associated bacteria, although living in physical proximity, are less likely to establish mutual exclusion processes, especially toward the end of the epipelagic zone. Bacteria that live on particles are known to harbor genomic features devoted to competition, e.g., type VI secretion systems and antibiotics (Dang and Lowell, 2016). Our data suggest that those traits might not necessarily be expressed under carbon limiting conditions in the deeper layers of the photic zone (Giering et al., 2014). However, it must be taken into account that our analysis was based on 16S amplicon sequencing of the rRNA gene and therefore do not provide any information on the activity of bacterial cells collected on the filters.

### Distribution of micro-eukaryotes and their co-occurrences with bacterioplankton

Bacterioplankton widely interact with phytoplankton (Amin et al., 2012, 2015; Lima-Mendez et al., 2015), and provide it with several compounds, like reduced forms of sulfur like DMSP (Tripp et al., 2008; Moran et al., 2012), polysaccharides (Teeling et al., 2012; Buchan et al., 2014), vitamins (Wagner-Döbler et al., 2010; Amin et al., 2012) and growth hormones (Amin et al., 2015). From the small (SPA) and large (LPA) particles we retrieved different groups of eukaryotic microalgae based on the plastidial 16S rRNA gene. Eukaryotic 16S rRNA sequences that are amplified with universal bacterial primers are usually discarded in most environmental studies, but they actually provide taxonomically useful information. We show that sequences retrieved with the bacterial primers F807 and R1050 (Bohorquez et al., 2012) cover almost 70% of the marine eukaryotic photosynthetic lineages of the PhytoREF database (Decelle et al., 2015) offering the opportunity to investigate the distribution of eukaryotic microalgae. However, interpretation must take into account the primer coverage and the issue of 16S rRNA gene copy number, which varies across taxonomic groups. In our study, the two most abundant classes of eukaryotic microalgae identified were Prymnesiophyceae (haptophytes) and Bacillariophyceae (diatoms). These results are similar to previous studies that used other molecular tools (Kirkham et al., 2011, 2013; de Vargas et al., 2015) or measurement of accessory pigments via HPLC (Barlow et al., 2002). The distribution of these two classes of eukaryotic microalgae observed here is consistent with worldwide modeling based on accessory pigments (Liu et al., 2009). Thus, our analysis was able to detect accurately the main players of the photosynthetic micro-eukaryotic community.

Co-occurrence analysis among bacteria and phytoplankton showed no clear taxonomic pattern. Several bacterial taxa coexisted with different micro-eukaryotes. This finding sustains the competitive “lottery hypothesis” (Sale, 1979), according to which species that belong to the same ecological guild, and hence, have the same ecological function, colonize spatial niches by chance. Several studies have shown the absence of species-specific associations among bacteria and macro-algae (Campbell et al., 2015; Marzinelli et al., 2015; Dittami et al., 2016), confirming the lottery hypothesis. Furthermore, we observed that most of the correlations between bacteria and micro-eukaryotes were negative (~60%). Those findings indicate mutual exclusion between the two communities of the plankton. A previous study investigating planktonic community at the SPOT station (Chow et al., 2014) has shown that top-down processes accounted for roughly 30% of the total correlations. Many micro-eukaryotic algae can be mixotrophic and feed on bacteria (Hartmann et al., 2012, 2013). Studies on members of the roseobacter clade have shown that the relationship between micro-eukaryotes and bacteria can switch from a mutualistic toward a pathogenic stage during co-culture (Seyedsayamdost et al., 2011; Wang et al., 2014).

## Conclusion

Deep sequencing of the bacterioplankton revealed province, depth and filter size specific communities with little overlap in the epipelagic zone of the Atlantic Ocean. Network analysis indicated that there are more correlations in the free-living bacteria than in the communities associated to particles. Co-occurrence patterns were shown to be bathymetrically stratified with a reduction of associations in the deepest layer of the photic zone. Negative correlations were especially high in the upper layers of the photic zone and decreased with depth and particle size. Eukaryotic microalgae-bacteria network analysis showed that there was no specific taxonomic association between bacterial taxa and micro-eukaryotic algae. Understanding how bacteria interact across space and time with other parts of the biota is essential in order to predict the adaptation and evolution of the ocean microbiome in the framework of climate change.

## Author contributions

MM isolated the DNA, IP, DP, MW provided the method for sequencing and constructed the amplicon libraries and RJ performed bioinformatics analysis. MM, JT, and ZD analyzed the data. TB analyzed the oceanographic data, JD contributed to the data analysis. HG, MW, and MS measured environmental parameters. IW and HW collected the samples during the oceanographic cruise. MS organized the oceanographic cruise. IW supported and supervised the research. MM and IW wrote the manuscript, all authors reviewed the manuscript.

### Conflict of interest statement

The authors declare that the research was conducted in the absence of any commercial or financial relationships that could be construed as a potential conflict of interest.

## Supplementary material

Table S1 **Environmental metadata and geographical positions**. Geographical location of sampling stations, environmental parameters, and factors used for multivariate statistics (province, depth layer) are reported.

Table S2 **Taxonomic placement of ambiguous reads**. Sequences that could not be assigned at the domain and phylum level were manually reassigned, when possible, via phylogenetic analysis. Results from the original taxonomic classification and the reassignment can be found in the sheet “SINA and curated classification,” while the BLAST results against the SILVA database 119 NR are reported in “BLAST ambiguous sequences.”

Table S3 **Sequencing results and taxonomic affiliation of OTUs**. Numbers of raw reads and bacterial sequences are listed, together with information about the relative abundance of each OTU within the three bacterial communities FL, SPA, and LPA and the eukaryotic microalgae SP and LP. For each OTU abundance data, sequence, and taxonomic information according to the SILVA 119 NR database are reported.

Table S4 **Networks data for the three size fractions of the plankton along the water column**. For each of the 15 networks calculated as described in Materials and Methods the original data are reported. Only statistical significant (*p* < 0.05) and strong correlations *R* > 0.6 are listed.

Table S5 **Number of indirect correlations with environmental parameters removed**.

Table S6 **Percentage of OTUs classified at different taxonomic levels**.

Table S7 **Coverage of primers F805 and R1050 of the marine photosynthetic lineages**. Chloroplast 16S of all the marine photosynthetic eukaryotes were extracted from the PhytoREF database and in silico investigated for the coverage of the primers.

Table S8 **Topological coefficients of the depth stratified networks for the FL community**.

Table S9 **Topological coefficients of the of the depth stratified networks for the SPA community**.

Table S10 **Topological coefficients of the of the depth stratified networks for the LPA community**.

Table S11 **Number of average neighbors for all size fractions of the plankton (FL, SPA, and LPA) without negative correlations**.

Table S12 **Percentage of positive and negative correlations among OTUs and among OTUs and environmental parameter for the depth stratified FL networks**.

Table S13 **Percentage of positive and negative interactions among OTUs and among OTUs and environmental parameter for the depth stratified SPA networks**.

Table S14 **Percentage of positive and negative interactions among OTUs and among OTUs and environmental parameter for the depth stratified LPA networks**.

Table S15 **Topological coefficients for the depth stratified co-occurrence networks between FL bacteria and micro-eukaryotes collected on the 8 μm membranes**.

Table S16 **Topological coefficients for the depth stratified co-occurrence networks between LPA bacteria and micro-eukaryotes collected on the 8 μm membranes**.

Table S17 **Percentage of positive and negative correlations between FL bacteria and LP micro-eukaryotes and LPA bacteria with LP micro-eukaryotes**.

Figure S1 **Phylogenetic analysis of ambiguously assigned sequences**. Sequences that could not be taxonomically assigned further than domain level were manually inspected. Reference sequences were downloaded from SILVA NR 119 and the neighbor-joining method was used for tree construction.

Figure S2 **Cumulative dominance plots:** After pre-treatment of the OTUs abundances as reported in materials and methods, a dominance plot was calculated for each of the three size fractions of the plankton: FL, SPA, and LPA. The first 10 most abundant OTUs are reported for each plot (x-axis) and their relative cumulative contribution (y-axis).

Figure S3 **Relative abundance of small particle associated (SPA) marine bacterial clades and genera for the six oceanographic provinces across the whole photic zone:** Relative abundance of OTUs classified at the genus and clade level was averaged for the six oceanographic provinces in three depth layers. Each line of the heat map represents a taxon, and its higher taxonomic rank (phylum or class) is indicated by the color key.

Figure S4 **Relative abundance of large particle associated (LPA) marine bacterial clades and genera for the six oceanographic provinces across the whole photic zone:** Relative abundance of OTUs classified at the genus and clade level was averaged for the six oceanographic provinces in three depth layers. Each line of the heat map represents a taxon, and its higher taxonomic rank (phylum or class) is indicated by the color key.
