# Supplementary material for: Co-occurrence Analysis of Microbial Taxa in the Atlantic Ocean Reveals High Connectivity in the Free-Living Bacterioplankton
Source: Front Microbiol. 2016 May 6;7:649. doi: 10.3389/fmicb.2016.00649 (PMC4858663; doi:10.3389/fmicb.2016.00649)
Supplement: Table S5 — Number of indirect correlations with environmental parameters removed. [file Table5-17.DOCX]

Table S5: Number of indirect correlations with environmental parameters: removed according to the methods described in material and methods section.

| **Network** | **All correlations with environmental triplets** | **All correlations after removal of environmental triplets** | **Number of environmental triplets** |
| --- | --- | --- | --- |
| **FL_20** | 1563 | 1541 | 22 |
| **FL_40** | 1521 | 1519 | 2 |
| **FL_60** | 1480 | 1458 | 22 |
| **FL_100** | 1412 | 1411 | 1 |
| **FL_200** | 852 | 852 | 0 |
| **SPA_20** | 1595 | 1439 | 156 |
| **SPA_40** | 1557 | 1474 | 83 |
| **SPA_60** | 969 | 969 | 0 |
| **SPA_100** | 1110 | 1105 | 5 |
| **SPA_200** | 345 | 345 | 0 |
| **LPA_20** | 1223 | 965 | 258 |
| **LPA_40** | 1183 | 1129 | 54 |
| **LPA_60** | 918 | 869 | 49 |
| **LPA_100** | 747 | 745 | 2 |
| **LPA_200** | 258 | 258 | 0 |

Table S6: Percentage of OTUs classified at different taxonomic levels. Percentages were calculated on the basis of the total number of OTUs that passed through the pipeline (see material and methods).

| Taxonomic level | FL | SPA | LPA |
| --- | --- | --- | --- |
| Domain (%) | 90.55 | 83.8 | 67.23 |
| Phylum (%) | 90.55 | 83.48 | 66.95 |
| Class (%) | 84.96 | 75.7 | 63.24 |
| Order (%) | 68.18 | 69.15 | 59.25 |
| Family (%) | 35.31 | 42.99 | 36.75 |
| Genus (%) | 15.73 | 23.98 | 19.37 |
| Clade/division (%) | 65.38 | 45.79 | 36.46 |
| Unclassified/Archaea (%) | 9.45 | 10.6 | 11.98 |
| Chloroplast 16S (%) | 0 | 5.60 | 20.79 |

Table S7: Coverage of primers F805 and R1050 of the marine photosynthetic lineages. Chloroplast 16S of all the marine photosynthetic eukaryotes were extracted from the PhytoREF database and *in silico* investigated for the coverage.

| **Taxonomic group** | **Coverage %** | **Sequences included in PhytoREF** | **Sequences matched** |
| --- | --- | --- | --- |
| **Diatoms** | 92 | 1094 | 1012 |
| **Chlorophyta** | 55 | 653 | 362 |
| **Eustigmatophyceae** | 52 | 312 | 164 |
| **Rhodophyta** | 93 | 161 | 150 |
| **Cryptophyceae** | 87 | 126 | 110 |
| **Prasinophyceae** | 95 | 82 | 78 |
| **Trebouxiophyceae** | 50 | 146 | 73 |
| **Euglenophyceae** | 24 | 288 | 70 |
| **Ulvophyceae** | 70 | 82 | 58 |
| **Dictyochophyceae** | 90 | 60 | 54 |
| **Nephroselmidophyceae** | 94 | 51 | 48 |
| **Phaeophyceae** | 91 | 46 | 42 |
| **Bangiophyceae** | 89 | 39 | 35 |
| **Chlorophyceae** | 43 | 76 | 33 |
| **Chrysophyceae** | 88 | 35 | 31 |
| **Haptophyta** | 8 | 369 | 31 |
| **Chlorodendrophyceae** | 91 | 21 | 23 |
| **Glaucocystophyceae** | 90 | 20 | 18 |
| **Raphidophyceae** | 38 | 26 | 10 |
| **Pinguiophyceae** | 100 | 8 | 8 |
| **Porphyridiophyceae** | 100 | 6 | 6 |
| **Rappemonads** | 100 | 6 | 6 |
| **Mamiellophyceae** | 0 | 129 | 0 |
| **Dinophyceae** | 11 | 34 | 4 |
| **Chlorarachniophyceae** | 100 | 20 | 20 |
| **Bolidophyceae** | 93 | 16 | 15 |
| **Apicomplexans** | 39 | 33 | 13 |
| **Pedinophyceae** | 94 | 18 | 17 |

Table S8: Topological coefficients of the depth stratified networks for the FL community

|  | **20 m** | **40 m** | **50-80 m** | **85-120 m** | **140-200 m** |
| --- | --- | --- | --- | --- | --- |
| **Number of edges** | 1538 | 1500 | 1443 | 1410 | 849 |
| **Number of nodes** | 134 | 141 | 151 | 157 | 130 |
| **Clustering coefficient** | 0.56 | 0.61 | 0.54 | 0.53 | 0.48 |
| **Network radius** | 1 | 4 | 4 | 4 | 4 |
| **Network centralization** | 0.29 | 0.28 | 0.23 | 0.2 | 0.19 |
| **Shortest path** | 17294 (97%) | 19740 (100%) | 23256 (100%) | 24492 (100%) | 16770 (100%) |
| **Characteristic path length** | 2.6 | 2.6 | 2.7 | 2.7 | 2.8 |
| **Avg number of neighbors** | 23 | 21.2 | 18.7 | 18 | 13.1 |
| **Network density** | 0.17 | 0.15 | 0.12 | 0.12 | 0.1 |
| **Network heterogeneity** | 0.88 | 0.79 | 0.71 | 0.69 | 0.71 |
| **Network diameter** | 8 | 7 | 8 | 8 | 6 |
| **Connected component** | 2 | 1 | 1 | 1 | 1 |

Table S9: Topological coefficients of the of the depth stratified networks for the SPA community

|  | **20 m** | **40 m** | **50-80 m** | **85-120 m** | **140-200 m** |
| --- | --- | --- | --- | --- | --- |
| **Number of edges** | 1437 | 1474 | 967 | 1105 | 344 |
| **Number of nodes** | 117 | 118 | 160 | 164 | 110 |
| **Clustering coefficient** | 0.57 | 0.57 | 0.48 | 0.54 | 0.43 |
| **Network radius** | 4 | 1 | 1 | 4 | 1 |
| **Network centralization** | 0.32 | 0.31 | 0.18 | 0.14 | 0.15 |
| **Shortest path** | 12572 (100%) | 12220 (88%) | 23568 (100%) | 26732 (100%) | 11558 (96%) |
| **Characteristic path length** | 2.5 | 2.4 | 3.3 | 2.9 | 3.4 |
| **Avg number of neighbors** | 24.6 | 25 | 12.1 | 13.4 | 6.3 |
| **Network density** | 0.21 | 0.21 | 0.07 | 0.08 | 0.06 |
| **Network heterogeneity** | 0.82 | 0.83 | 0.76 | 0.64 | 0.65 |
| **Network diameter** | 8 | 8 | 10 | 8 | 9 |
| **Connected component** | 1 | 4 | 4 | 1 | 2 |

Table S10: Topological coefficients of the of the depth stratified networks for the LPA community

|  | **20 m** | **40 m** | **50-80 m** | **85-120 m** | **140-200 m** |
| --- | --- | --- | --- | --- | --- |
| **Number of edges** | 965 | 1125 | 867 | 744 | 243 |
| **Number of nodes** | 137 | 164 | 162 | 181 | 110 |
| **Clustering coefficient** | 0.44 | 0.45 | 0.44 | 0.42 | 0.35 |
| **Network radius** | 1 | 4 | 1 | 1 | 1 |
| **Network centralization** | 0.28 | 0.28 | 0.17 | 0.13 | 0.1 |
| **Shortest path** | 18092 (97%) | 26732 (100%) | 23880 (100%) | 31864 (97%) | 9140 (76%) |
| **Characteristic path length** | 2.8 | 3 | 3.3 | 3.7 | 4.5 |
| **Avg number of neighbors** | 14.1 | 13.7 | 10.7 | 8.2 | 4.4 |
| **Network density** | 0.1 | 0.08 | 0.07 | 0.04 | 0.04 |
| **Network heterogeneity** | 0.91 | 0.9 | 0.79 | 0.77 | 0.82 |
| **Network diameter** | 7 | 7 | 10 | 10 | 13 |
| **Connected component** | 2 | 1 | 4 | 2 | 7 |

Table S11: Number of average neighbors for all size fractions of the plankton (FL, SPA and LPA) without negative correlations

|  | **FL** | **SPA** | **LPA** |
| --- | --- | --- | --- |
| **20 m** | 14.3 | 13,4 | 7.8 |
| **40 m** | 12.4 | 13.4 | 8,3 |
| **50-80 m** | 11,7 | 9 | 7.3 |
| **85-120 m** | 11.1 | 9.1 | 5.8 |
| **140-200 m** | 8 | 4.6 | 3.7 |

Table S12: Percentage of positive and negative correlations among OTUs and among OTUs and environmental parameter for the depth stratified FL networks.

|  | **20 m** | **40 m** | **50-80 m** | **85-120 m** | **140-200 m** |
| --- | --- | --- | --- | --- | --- |
| **Positive interactions (%)** | 62.2 | 58.5 | 62.8 | 61.5 | 60.8 |
| **Negative interactions (%)** | 37.8 | 41.5 | 37.2 | 38.5 | 39.2 |
| **Interactions among OTUs (%)** | 98.3 | 98.4 | 97.6 | 99.3 | 96.9 |
| **Positive interactions among OTUs (%)** | 62.4 | 58.5 | 63 | 61.8 | 61.8 |
| **Negative interactions among OTUs (%)** | 37.6 | 41.5 | 37 | 38.2 | 38.2 |
| **Interactions with environmental parameters (%)** | 1.7 | 1.6 | 2.4 | 0.7 | 3.1 |
| **Positive interactions with environmental parameters (%)** | 55.6 | 54.2 | 55.9 | 20 | 27 |
| **Negative interactions with environmental parameters (%)** | 44.4 | 45.8 | 44.1 | 80 | 73 |

Table S13: Percentage of positive and negative interactions among OTUs and among OTUs and environmental parameter for the depth stratified SPA networks.

|  | **20 m** | **50 m** | **50-80 m** | **85-120 m** | **140-200 m** |
| --- | --- | --- | --- | --- | --- |
| **Positive interactions (%)** | 54.6 | 53.5 | 74.1 | 67.8 | 71.5 |
| **Negative interactions (%)** | 45.4 | 46.5 | 25.9 | 32.2 | 28.5 |
| **Interactions among OTUs (%)** | 97.2 | 97.6 | 99.2 | 98.3 | 96.8 |
| **Positive interactions among OTUs (%)** | 54.3 | 53.1 | 74.2 | 68.5 | 72.1 |
| **Negative interactions among OTUs (%)** | 45.7 | 46.9 | 25.8 | 31.5 | 27.9 |
| **Interactions with environmental parameters (%)** | 2.8 | 2.4 | 0.8 | 1.7 | 3.2 |
| **Positive interactions with environmental parameters (%)** | 65 | 69.5 | 62.5 | 26.3 | 55.6 |
| **Negative interactions with environmental parameters (%)** | 35 | 30.5 | 37.5 | 73.7 | 45.4 |

Table S14: Percentage of positive and negative interactions among OTUs and among OTUs and environmental parameter for the depth stratified LPA networks.

|  | **20 m** | **50 m** | **50-80 m** | **85-120 m** | **140-200 m** |
| --- | --- | --- | --- | --- | --- |
| **Positive interactions (%)** | 55 | 61.1 | 68.9 | 71.2 | 88.1 |
| **Negative interactions (%)** | 45 | 38.9 | 31.1 | 28.8 | 11.9 |
| **Interactions among OTUs (%)** | 95.6 | 98 | 97.5 | 97.7 | 95.5 |
| **Positive interactions among OTUs (%)** | 54.6 | 60.7 | 69.3 | 71.9 | 87.9 |
| **Negative interactions among OTUs (%)** | 45.4 | 39.3 | 30.7 | 28.1 | 12.1 |
| **Interactions with environmental parameters (%)** | 4.4 | 2 | 2.5 | 2.3 | 4.5 |
| **Positive interactions with environmental parameters (%)** | 61.3 | 78.3 | 86.4 | 41.2 | 91 |
| **Negative interactions with environmental parameters (%)** | 38.7 | 21.7 | 13.6 | 58.8 | 9 |

Table 15: Topological coefficients for the depth stratified co-occurrence networks between FL bacteria and micro-eukaryotes collected on the 8µm membranes.

|  | **20 m** | **40 m** | **50-80 m** | **85-120 m** |
| --- | --- | --- | --- | --- |
| **Number of edges** | 611 | 708 | 433 | 485 |
| **Number of nodes** | 129 | 159 | 177 | 186 |
| **Clustering coefficient** | 0 | 0 | 0 | 0 |
| **Network radius** | 4 | 6 | 1 | 6 |
| **Network centralization** | 0.39 | 0.32 | 0.2 | 0.2 |
| **Shortest path** | 16512 (100%) | 25122 (100%) | 30108 (100%) | 34110 (100%) |
| **Characteristic path length** | 3 | 3.3 | 4.1 | 3.8 |
| **Avg number of neighbors** | 9.5 | 8.9 | 4.9 | 5.2 |
| **Network density** | 0.07 | 0.06 | 0.03 | 0.03 |
| **Network heterogeneity** | 1.2 | 1.2 | 1.2 | 1.1 |
| **Network diameter** | 9 | 11 | 13 | 11 |
| **Connected component** | 1 | 1 | 2 | 1 |

Table 16: Topological coefficients for the depth stratified co-occurrence networks between LPA bacteria and micro-eukaryotes collected on the 8µm membranes.

|  | **20 m** | **40 m** | **50-80 m** | **85-120 m** |
| --- | --- | --- | --- | --- |
| **Number of edges** | 557 | 742 | 490 | 453 |
| **Number of nodes** | 166 | 208 | 198 | 212 |
| **Clustering coefficient** | 0 | 0 | 0 | 0.17 |
| **Network radius** | 1 | 1 | 1 | 1 |
| **Network centralization** | 0.26 | 0.24 | 0.16 | 0.13 |
| **Shortest path** | 26734 (97%) | 41424 (96%) | 34428 (88%) | 43892 (98%) |
| **Characteristic path length** | 4.1 | 3.6 | 3.9 | 4.3 |
| **Avg number of neighbors** | 6.7 | 7.2 | 4.9 | 4.3 |
| **Network density** | 0.04 | 0.03 | 0.03 | 0.02 |
| **Network heterogeneity** | 1.2 | 1.2 | 1.1 | 1 |
| **Network diameter** | 14 | 9 | 9 | 12 |
| **Connected component** | 2 | 2 | 6 | 2 |

Table S17: Percentage of positive and negative correlations between FL bacteria and LP micro-eukaryotes and LPA bacteria with LP micro-eukaryotes

|  |  | 20 | 40 | 50-80 | 85-120 |
| --- | --- | --- | --- | --- | --- |
| FL | Positive correlations | 22 | 23.7 | 41,2 | 41.2 |
|  | Negative correlations | 68 | 64.3 | 58,8 | 58.6 |
| LPA | Positive correlations | 35.5 | 37.1 | 32.3 | 48.3 |
|  | Negative correlations | 64.5 | 62.9 | 63.7 | 51.7 |
