# Supplementary figures and images for: Co-occurrence Analysis of Microbial Taxa in the Atlantic Ocean Reveals High Connectivity in the Free-Living Bacterioplankton
Source: Front Microbiol. 2016 May 6;7:649. doi: 10.3389/fmicb.2016.00649 (PMC4858663; doi:10.3389/fmicb.2016.00649)

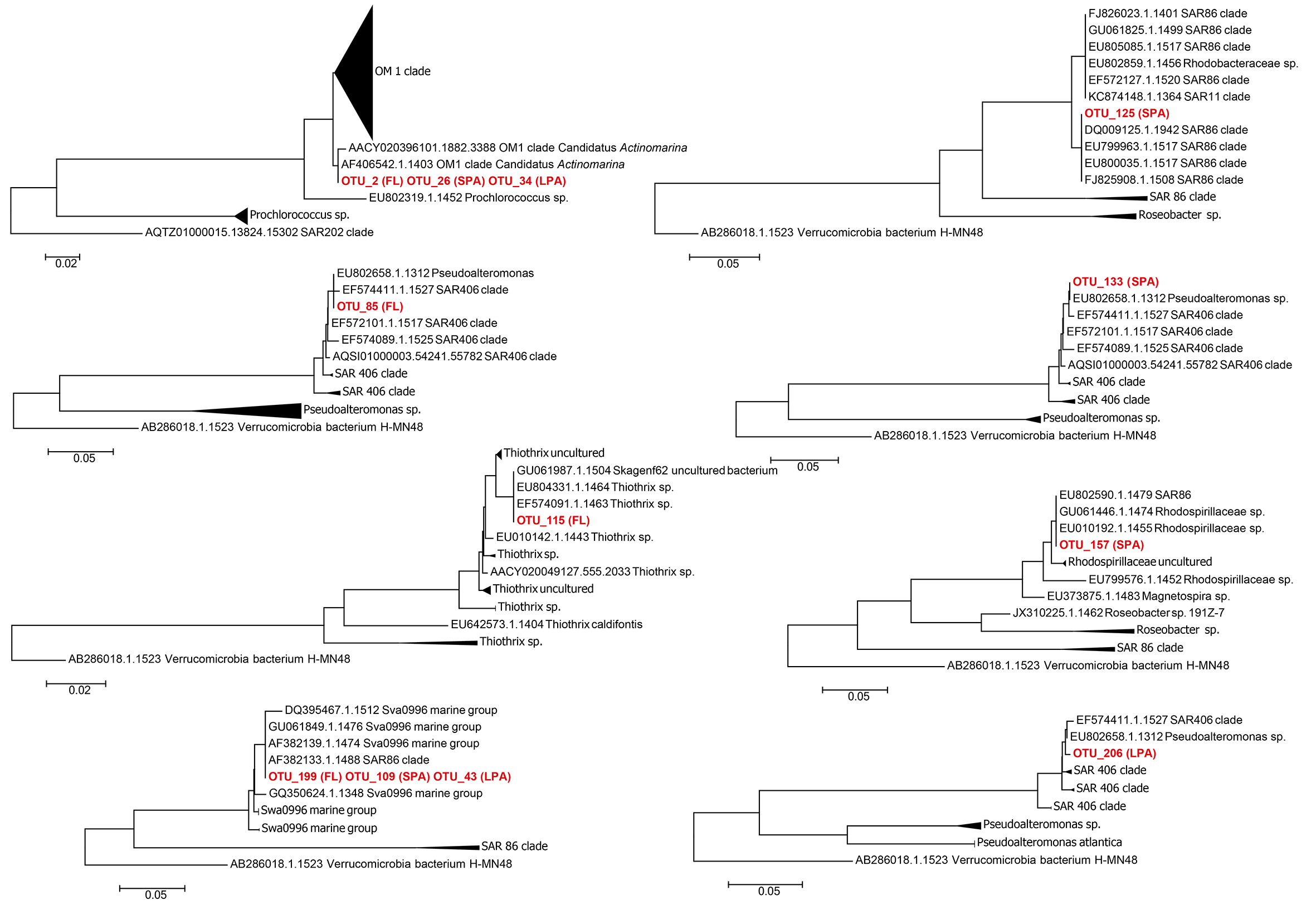

Supplement: Figure S1 — Phylogenetic analysis of ambiguously assigned sequences. Sequences that could not be taxonomically assigned further than domain level were manually inspected. Reference sequences were downloaded from SILVA NR 119 and the neighbor-joining method was used for tree construction. [file Image1.TIF]

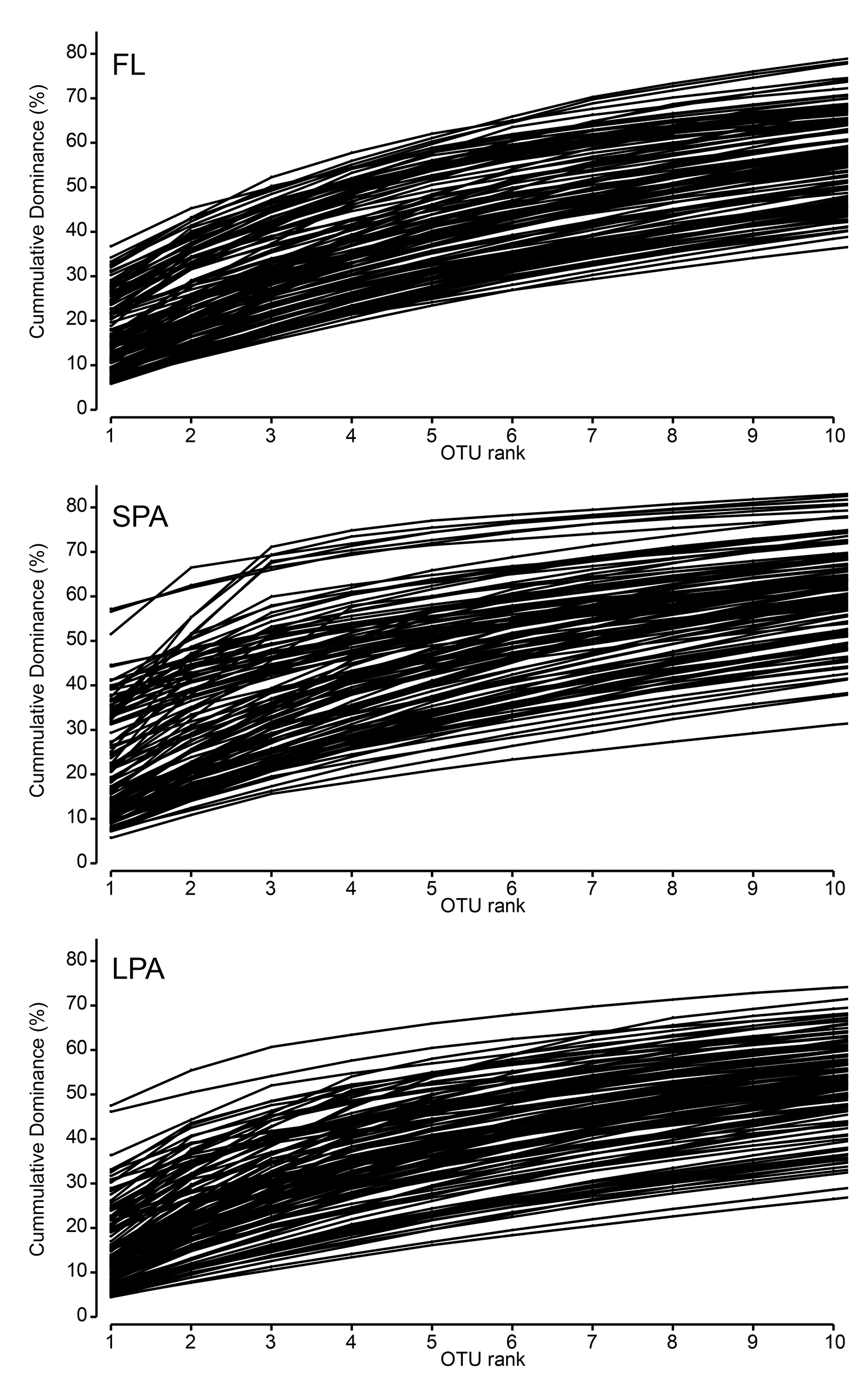

Supplement: Figure S2 — Cumulative dominance plots: After pre-treatment of the OTUs abundances as reported in materials and methods, a dominance plot was calculated for each of the three size fractions of the plankton: FL, SPA, and LPA. The first 10 most abundant OTUs are reported for each plot (x-axis) and their relative cumulative contribution (y-axis). [file Image2.TIF]

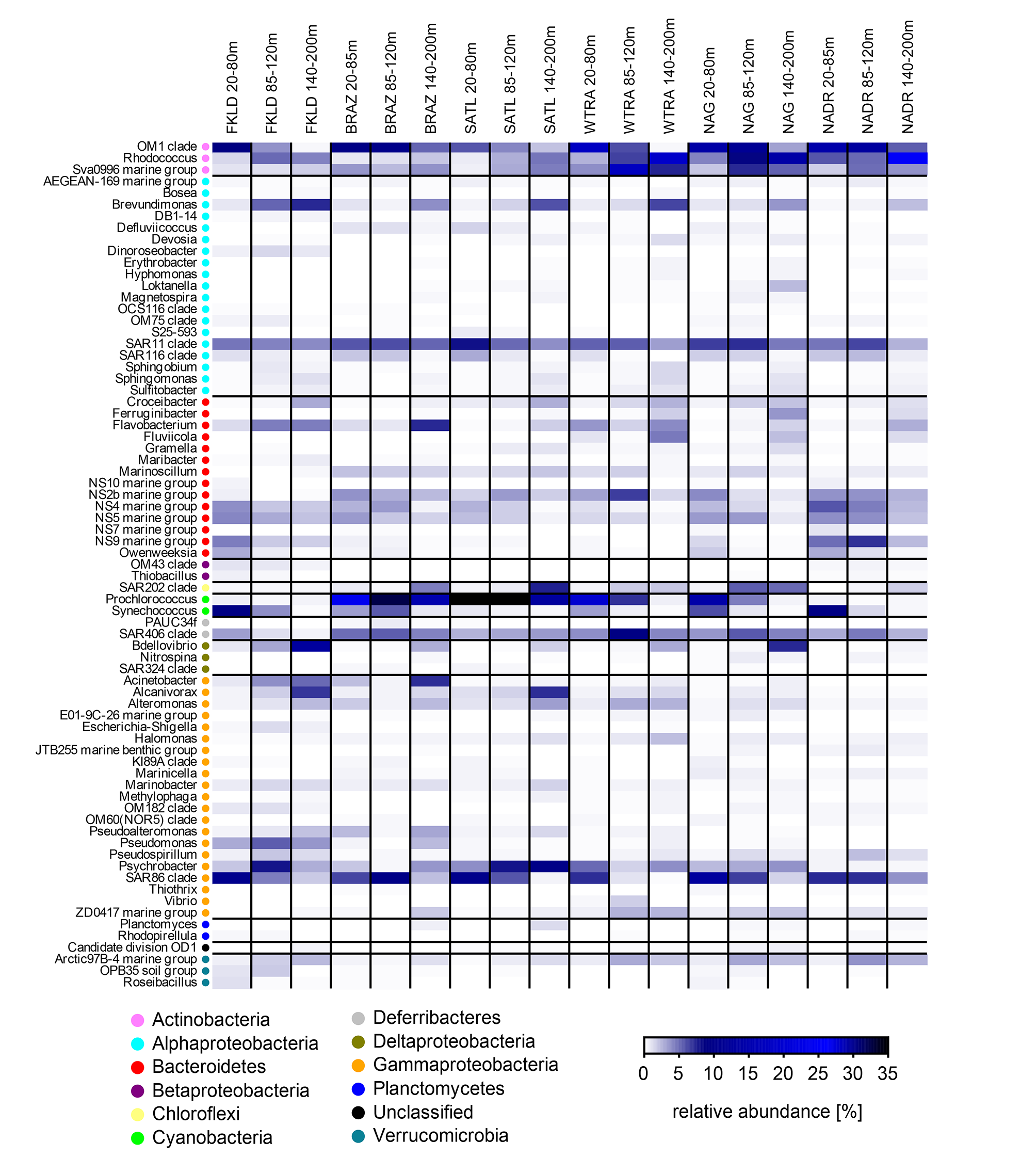

Supplement: Figure S3 — Relative abundance of small particle associated (SPA) marine bacterial clades and genera for the six oceanographic provinces across the whole photic zone: Relative abundance of OTUs classified at the genus and clade level was averaged for the six oceanographic provinces in three depth layers. Each line of the heat map represents a taxon, and its higher taxonomic rank (phylum or class) is indicated by the color key. [file Image3.TIF]

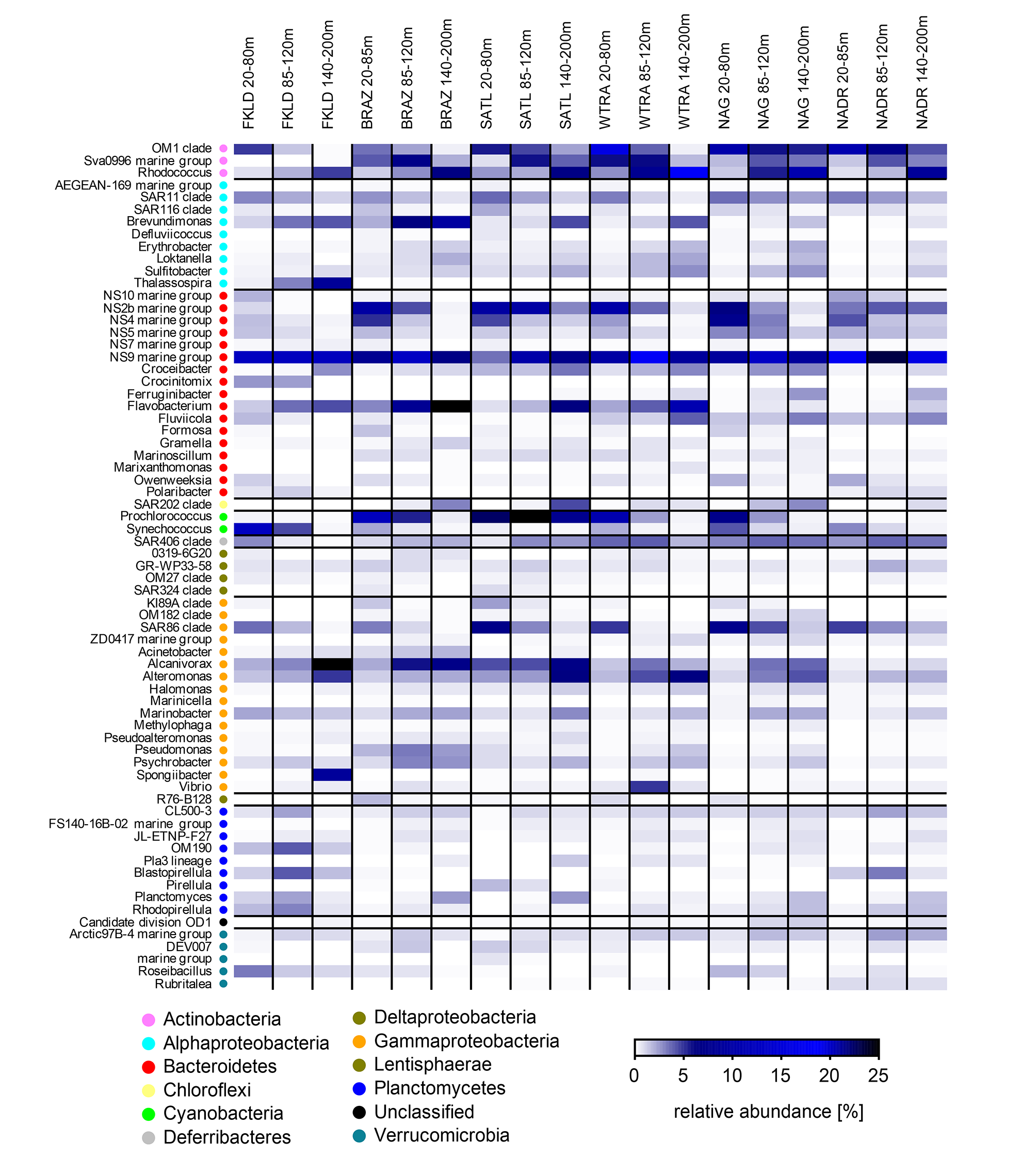

Supplement: Figure S4 — Relative abundance of large particle associated (LPA) marine bacterial clades and genera for the six oceanographic provinces across the whole photic zone: Relative abundance of OTUs classified at the genus and clade level was averaged for the six oceanographic provinces in three depth layers. Each line of the heat map represents a taxon, and its higher taxonomic rank (phylum or class) is indicated by the color key. [file Image4.TIF]
